# Intelligent Transport System Using Time Delay-Based Multipath Routing Protocol for Vehicular Ad Hoc Networks

**DOI:** 10.3390/s21227706

**Published:** 2021-11-19

**Authors:** Yashar Ghaemi, Hosam El-Ocla, Nitin Ramesh Yadav, Manisha Reddy Madana, Dheeraj Kurugod Raju, Vignesh Dhanabal, Vishal Sheshadri

**Affiliations:** Department of Computer Science, Lakehead University, Thunder Bay, ON P7B 5E1, Canada; yghemi@lakeheadu.ca (Y.G.); nramesh1@lakeheadu.ca (N.R.Y.); mmadana@lakeheadu.ca (M.R.M.); dkurugod@lakeheadu.ca (D.K.R.); vdhanaba@lakeheadu.ca (V.D.); vsheshad@lakeheadu.ca (V.S.)

**Keywords:** vehicle-to-vehicle (V2V), vehicular ad hoc network, routing, Intelligent Transportation System, transport

## Abstract

During the last decade, the research on Intelligent Transportation System (ITS) has improved exponentially in real-life scenarios to provide optimized transport network performance. It is a matter of importance that alert messages are delivered promptly to prevent vehicular traffic problems. The fact is an ITS system per se could be a part of a vehicular ad hoc network (VANET) which is an extension of a wireless network. In all sorts of wireless ad hoc networks, the network topology is subjected to change due to the mobility of network nodes; therefore, an existing explored route between two nodes could be demolished in a minor fraction of time. When it comes to the VANETs, the topology likely changes due to the high velocity of nodes. On the other hand, time is a crucial factor playing an important role in message handling between the network’s nodes. In this paper, we propose Time delay-based Multipath Routing (TMR) protocol that effectively identifies an optimized path for packet delivery to the destination vehicle with a minimal time delay. Our algorithm gives a higher priority to alert messages compared to normal messages. It also selects the routes with the short round-trip time (RTT) within the RTT threshold. As a result, our algorithm would realize two goals. Firstly, it would speed up the data transmission rate and deliver data packets, particularly warning messages, to the destination vehicle promptly and therefore avoid vehicular problems such as car accidents. Secondly, the TMR algorithm reduces the data traffic load, particularly of the normal messages, to alleviate the pressure on the network and therefore avoids network congestion and data collisions. This, in turn, lessens the packets’ retransmissions. To demonstrate the effectiveness of the proposed protocol, the TMR has been compared with the other protocols such as AOMDV, FF-AOMDV, EGSR, QMR, and ISR. Simulation results demonstrate that our proposed protocol proves its excellent performance compared to other protocols.

## 1. Introduction

In recent years, Intelligent Transportation Systems (ITSs) in Vehicular ad hoc networks (VANETs) have become a popular and vital research topic in the transportation industry [1]. ITS is an advanced application that facilitates nodes’ mobility in a network at high velocity. In ITS, vehicles interact and communicate with each other to deliver messages for various purposes such as alerts, traffic jams, and accident zones on the road. Vehicular ad hoc networks are established with mainly two types of communication, i.e., vehicle-to-vehicle (V2V) communication and vehicle-to-infrastructure (V2I) communication [2]. V2V communication is used among the deployed vehicles with On-Board Units (OBU) in the system. V2I communication is used for the interaction between vehicles and Road-Side Units (RSU) [3]. Both V2V and V2I communications consist of two types of messages, normal messages and emergency messages. The normal messages are sent to the RSU and other vehicles in the same range, and these messages include the speed and Global Positioning System (GPS) information. The emergency messages contain collision details and must be sent to other responders to act promptly. As a result, the accident notification is sent before the normal message to the concerned destination vehicle. VANET communicates with high-speed moving nodes and alongside RSU where packet delivery should be maximized with the least tolerance for data loss [4,5]. In the event of an accident, the data must be sent to all nodes in the network as soon as possible since time plays a major role in avoiding vehicle collisions, particularly on the highway. A multipath routing protocol is capable of handling high loads of data, balancing the data traffic, and managing time better than a single path routing protocol. In the case of having a node or link failure, Multipath protocols provide alternative routes without going through the route discovery phase and this enhances the network performance compared to the single route mechanisms such as the Ad-hoc On-demand Distance Vector (AODV) protocol. In [6], the authors describe VANETs as being categorized as a sub of mobile ad hoc networks (MANETs). Routing in VANETs must be able to improve the efficiency of the traffic and provide an infotainment facility as well.

When the network undergoes excessive data traffic problems such as data congestion and packet collisions, the data transmission time increases. As a result, this would magnify the possibility of having vehicle traffic problems and car accidents when warning messages are not arriving at their destinations on time. It should be noted that data congestion and collision are two reasons for having packet loss in a wireless network in addition to random loss. In all these cases, data packet retransmission increases the traffic load on the network and hence the chance of having more traffic problems [7]. This, in turn, would degrade the performance in terms of the end-to-end delay and the throughput leading to vehicle traffic problems that should be avoided.

Based on the minimum number of hops, Ad hoc On-demand Multipath Distance Vector (AOMDV) provides alternative routes in the case of having a channel disconnect. However, it does not consider the status of the nodes in the sense of having traffic problems. In this paper, we propose a centralized ITS where vehicles can send data to RSU, which in turn would be conveyed to other vehicles. RSU will use our TMR protocol for data communication in an infrastructure-to-vehicle (I2V) mode in case of emergency messages. These messages are broadcast to all vehicles on the road to avoid critical incidents without going through the route discovery phase and this, in turn, reduces the time required for data transmission. The main aim of TMR is to deliver data messages by selecting the most optimized path in the network. TMR is a reactive protocol where RTT measurement is utilized to select the shortest path. Whenever the RSU needs to send a message to the destination vehicle, the optimized route is found based on the least RTT to transmit a message to the destination. Minimum RTT implies passing through nodes with the least traffic data. Additionally, the TMR algorithm lessens the amount of data packets to avoid network congestion and data collisions of data packets. Therefore, TMR will speed up the data transmission needed particularly to be less than the vehicle speed and hence avoid any delay of the alert messages. This would certainly alleviate the possibility of vehicle traffic problems. In doing this, a dynamic threshold should be met to find a route; otherwise, RSU should wait a backoff time before repeating the discovery phase, and hence the intermediate vehicles are getting closer with less traffic. Therefore, our proposed routing TMR algorithm can achieve the following contributions:

- Prioritize ITS emergency messages compared to normal packets. As a result, deliver both emergency warning and data packets in the VANET environment on time to avoid vehicular traffic problems;
- Select the shortest route that has the minimum time delay and, therefore, speed up the data transmission;
- TMR uses the minimum RTT to select the optimized route, and as a result, it can adapt to the topological change;
- Our protocol reduces the traffic problems by using a threshold value where it should be higher than the RTT value to add its route to the efficient routes array;
- Minimize the data traffic load by reducing the retransmission data packets and therefore enhance the network performance.

The analysis of TMR proves promising results and works better under different network performance metrics such as end-to-end delay, packet delivery ratio, packet loss ratio, throughput, and routing overhead compared to other protocols like AOMDV, Energy Efficient Multipath Routing Protocol for Mobile Ad-Hoc Network Using the Fitness Function (FF-AOMDV), Q-learning based Multi-objective optimization Routing protocol (QMR), Efficient Geographic aware Source Routing (EGSR), and Improved Road Segment-Based Geographical Routing (ISR).

The remaining sections of the paper are structured as follows: Section 2 presents a literature review. The problem has been stated, and the solution has been proposed in Section 3. Section 4 explains the methodology and Section 5 contains simulation setup, simulation results, and analysis of results. Section 6 is designated to the analysis of the complexity of the algorithm, which is followed by the articulated conclusion in Section 7.

## 2. Literature Review

A variety of algorithms and protocols have been proposed over the years to improve the data communication performance in VANETs. To overcome different challenges in VANET, various researchers proposed and improved different approaches.

### 2.1. Topological Approach

In [8], the authors proposed a new protocol named load balancing maximal minimal nodal residual energy ad hoc on-demand multipath distance vector routing protocol (LBMMRE-AOMDV) which is an extended version of the AOMDV protocol. The protocol consists of two phases. The first generates disjoint link paths and maintains them in case of having one or more path failures. The second phase balances the data load among the generated link-disjoint paths. Through these phases, the protocol evaluates the generated paths to determine the maximal nodal residual energy and the actual number of packets that can be transmitted over that path without depleting the nodes’ energy. Results achieve better performance in terms of packet delivery ratio and energy consumption while taking into account the number of dead nodes [9]. However, it suffers from a long end-to-end delay. Thus, the authors recommend using this protocol in applications such as banking and online shopping rather than generic applications.

In [10], the authors presented a hybrid protocol that is based on the AODV algorithm using fuzzy logic named An Enhanced Hybrid Routing Protocol in Vehicular Ad-hoc Networks (TIHOO) to restrict the phase of route discovery. This algorithm is efficient and improves performance. However, it is rather a complex algorithm and unrealistic as it obviously consumes energy and therefore reduces the node’s lifetime.

On the other hand, the authors in [11] focused on reducing energy consumption using FF-AOMDV protocol with dragonfly topology. FF-AOMDV uses the fitness function to find the optimal multipath routing. It has been proved that the FF-AOMDV algorithm produces better results in terms of energy consumption, PDR, throughput, end-to-end delay, and routing overhead ratio when compared to AOMDV and AOMR-LM (Ad-hoc On-Demand Multipath Routing with Life Maximization). However, the fitness function spends a long processing time and therefore enlarges the end-to-end delay.

In [12], the authors presented a traffic-aware routing protocol in VANET by introducing multi-objective auto-regressive whale optimization (ARWO) algorithm. ARWO selects the best path from multiple paths by considering multiple objectives such as end-to-end delay, link lifetime, and node distance in the fitness function. However, it suffers from network overload when there is high traffic and congestion on the routes since all the vehicles try to choose the best path to reach the destination.

In [13], Fault-Tolerant Disjoint Multipath Distance Vector Routing Algorithm (FD-AOMDV) was introduced. This algorithm finds the shortest path based on the residual energy of the intermediate nodes at the expense of the number of nodes of that selected path and, therefore, the transmission delay.

In [14], the authors presented a novel V2V-enabled resource allocation scheme based on cellular vehicle-to-everything (C-V2X) technology to improve the reliability and latency of VANETs. The main challenge with this cellular-based V2V technique is how to allocate spectrum resources and broadcast opportunities properly in the V2V communication, so the network performance can be improved without causing disturbing interference to cellular users.

In [15], a hierarchical failure detection mechanism based on the architecture of VANETs was suggested. The failure detector can adapt to the dynamic network conditions and meet the different Quality of Service (QoS) requirements of multiple applications in VANETs. By sharing messages among vehicles, communication link failures can be overcome, and detection accuracy is further improved. The major shortcoming of this mechanism is its routing overhead which increases as the number of detection messages increases.

In [16], the author proposed QMR routing protocol, a method based on Q-learning with a multi-objective optimization mechanism. In this protocol, the author tries to take advantage of both proactive and reactive routing protocols to optimize the delay and the efficiency of the network. Each node in the network is equipped with GPS. Based on the measured distance and residual energy, each node can determine to forward the data packets. This protocol imposes a high load of routing overhead.

In [17,18], it was proposed a method that set a service-level agreement (SLA) together with the energy cooperation for road planning and to select the quickest route for ambulance vehicles. In VANETs, energy is not the main concern as the battery is a reasonable source of power. In addition, the time complexity is relatively high which does not suit critical applications such as an ambulance.

The main drawback of the topological approach is that mobility is an overlooked challenge in the design of routing protocols. In topological routing protocols, the network is being considered as a graph of nodes in terms of vehicles and edges, which are the connectivity between adjacent nodes. Since the mobility in VANETs is fast, the constructed graph is subjected to change frequently. With the occurrence of a link failure, the graph needs to be reconstructed, which imposes a high cost in terms of routing overhead and end-to-end delay.

### 2.2. Road and Traffic Awareness Approach

In [19], the authors proposed a Multi-metric Geographic Routing (M-GEDIR) algorithm to select the next hop. The next node vehicle is selected from the dynamic forwarding region based on the probability of the area being safe or unsafe. It is a V2V communication where the roadside unit is not considered. This is an unrealistic approach as the processing time is long to determine the optimal path.

In [20], a mechanism was developed based on a combination of the intersection-based routing technique with the shortest path-based traffic light-aware routing protocol. The protocol was named Reliable Path Selection and Packet Forwarding Routing Protocol (RPSPF) by authors. Data packets are transmitted based on the traffic patterns and the traffic light signals along the intersection. The protocol could provide a high delivery ratio and throughput but has connectivity issues like the end-to-end connectivity among vehicles will be broken when one node goes away, leaving the network. Thus, vehicles should reconnect to communicate.

In [21] an efficient Parked Vehicle Assistant Relay Routing (PVARR) algorithm was proposed to guarantee vehicle-to-vehicle communication in VANETs. Based on this algorithm, practical cooperation between stationary and moving vehicles prevents the network from having broadcast storms, increases resource utilization rate, and effectively diminishes load on the network. Results prove that the packet delivery ratio for the proposed protocol is high. This mechanism does not use RSU as intermediate nodes while the communication is rather running through parked vehicles in that network. Having parked vehicles on the road, particularly highways, is not always common, and therefore this protocol is not applicable if no parked vehicles are available on the road. On the contrary, using RSU can reduce the number of hops (because there is a range for each RSU), which simultaneously reduces the delay when compared to parked vehicles.

To enhance geographical source routing (GSR) protocol, research [22] proposed an efficient GSR (EGSR). This protocol has been designed mainly for urban areas, and it is assumed that every node in the network is equipped with a GPS and digital map. Additionally, it is assumed that the clocks of all nodes are synced. The proposed protocol uses the ant-colony algorithm to find the optimum path between nodes in the network. Each node can calculate the weight of the road segments by a small packet called ANT, which is generated by vehicles at the intersections. Based on the weight of every road segment, the sender selects the best road route to the destination. The road segment is constituted by distance and the delay between intersections. This protocol relies on the delay, which requires an accurate synchronization to be calculated. In addition, routing tables are updated using ANT packets generated in a timely fashion based on a constant value of $t_{ant}$. When $t_{ant}$ is short, routing overhead load is high, causing network instability and performance degradation.

In [23], it was introduced a new mechanism called Distance and Direction based Location aided routing (DD-LAR), which is an extended version of the existing Directional-Location Aided Routing (D-LAR) Protocol. In this protocol, the sender node first checks for the locational information of its nearby neighboring nodes in the request zone within its transmission range. The neighboring node that is positioned at the minimum angular deviation and a maximum distance from the sender node will be selected as the next-hop forwarding node. However, in case of having a conflict in which the node has the minimum angular deviation while not having the maximum distance, then the algorithm prefers the previous version because the relative distance of all the nodes from the source node is negligible. Moreover, the previous version (D-LAR) gives a better hop count in a dense environment like the city traffic conditions.

In [24], the authors proposed ISR protocol. This protocol uses segments for data transmission in urban VANET. The road map is divided into different segments, and routing takes place based on the information of the next segment. This protocol has been designed based on parameters such as node position information, direction, vehicular congestion density, and link quality between the communicating nodes. The protocol is highly dependent on GPS accuracy and availability. Therefore, in case of GPS absence, like tunnels, where having a network connection is crucial, the protocol does not function properly.

In the intersection-aware approach, the main challenge is its high end-to-end delay. This approach is practical to confront physical obstacles such as buildings. Data are forwarded through intersections which are selected one by one. As a result, this mechanism imposes a high processing delay. In return, a lower cost is generated in terms of routing overhead. On the other hand, protocols in the traffic density aware sub-category consider vehicular congestion as a metric to evaluate the connectivity [25]. However, a highly congested road is vulnerable to having data congestion problems.

### 2.3. Clustering Approach

The paper [26] presents a moving-zone-based architecture and routing protocol for data transmission in vehicle-to-vehicle communication. A head vehicle is assigned for each area, and it is in charge of managing the information of other member vehicles as well as the message dissemination. This mechanism does not work with vehicle-to-infrastructure (vehicle-to-RSU) communication because the captain vehicle itself acts as a centralized unit. Clusters are formed by vehicles having similar moving patterns. In each cluster, one vehicle is elected as the section captain. The captain node is responsible for managing other nodes’ information and handling data transmission. The sender node forwards its data to the captain. If the captain node detects that the receiver end is outside its zone, it forwards the data to a member node that is the nearest to the destination end. The chance of bottleneck occurrence for the captain node is high, which could result in having longer end-to-end delay and a higher chance of packet loss. Additionally, this scheme imposes high energy costs on the captain node.

To conquer the issues of the traffic-aware approach, authors in [27] proposed a delay-aware grid-based geographical clustering method. The novelty of this work is to address desirable performance in both scenarios of dense and spare vehicular congestion. Different backbone nodes compose backbone links in each street segment. Inter-grid and intra-grid mechanisms have been proposed to accommodate the data transmission in all traffic models. In addition to the dependency of protocol on online maps and GPS, the method requires complicated calculations.

In [28], the author proposed a clustering technique to transmit emergency messages such as road accidents with a minimum possible delay. This clustering technique handles broadcast storm problems to reduce network congestion. Simulation of this mechanism provides better results only at low speed. When the vehicle speed increases, it results in poor performance of the system as the network connectivity decreases.

Many clustering protocols overlook the road structure in their design. In [29], the proposed method tries to overcome the possible upcoming issues at the intersections related to change of direction and mobility speed with the assistance of RSU nodes. Two adjacent intersections and the corresponding road is considered as road segment. The connectivity of each road segment is studied based on the average end-to-end delay in both scenarios of high and low-density road traffic. In this paper, the sender node decides the next forwarding RSU positioned at a nearby intersection. Then the data load is forwarded to the next intersection, which is close to the destination node through intermediate nodes in the next adjacent road sections until it reaches the destination vehicle.

The protocols under the category of clustering mainly provide a high rate of packet delivery. Additionally, scalability is another feature of this group. These protocols can adapt to different environments with different predictable mobility patterns. However, clustering approach protocols increase the latency in consequence of the communications involved between the sender node and the cluster head, and this consumes a processing time.

### 2.4. Time Delay-Based Approach

In [30], authors propose a routing protocol named Congestion-aware Fibonacci Multipath Load Balancing (Congestion-aware FMLB). This protocol discovers routes based on the minimum RTT. The data packet distributes through different routes based on the Fibonacci sequence number; therefore, the route with the smallest RTT is used more often. The analysis of results in this paper shows the superiority of RTT over the number of hops routing mechanism. This protocol’s main issue is the high chance of finding lengthy routes due to the absence of a mechanism to restrict the route lengthiness. These lengthy routes participate in the practice of data transmission according to the load balancing mechanism.

An RTT-based probabilistic routing protocol for content-centric networking (CCN) known as a receiver-driven network architecture is proposed in [31]. This method selects the paths based on the message content; therefore; one packet may pass to multiple servers and accordingly the RTT measurement is not accurate.

Authors in [32] proposed a novel delay metric named sojourn time backlog (STB) employed with the back-pressure (BP) algorithm. The STBP protocol was introduced to overcome the problem of having long end-to-end delays in the BP scheme. STB considers queuing delay instead of queue length to select the routes. However, this method is yet not a loop-free routing method; therefore, the provided delay is unsuitable for real-time network applications.

In [33,34], two different routing protocols of Reliable Cluster-based Energy-aware Routing (RCER) protocol and load-balanced Multipath routing protocol with energy constraints (EE-LB-AOMDV) were proposed respectively to optimize the routes based on the three metrics of residual energy, hop count, and RTT. RCER mainly aims to maximize the network lifetime by clustering the network into the geographical segments based on the nodes’ residual energy and the node neighboring. This protocol is suited for a network with low nodes mobility to avoid frequent cluster reconstruction. EE-LB-AOMDV load balances the data over different discovered routes based on the route score to increase the lifetime of the network. Due to the absence of a restriction mechanism for the lengthiness of routes in this protocol, it is likely to send data through routes with high end-to-end delay.

An efficient routing algorithm in VANETs that considers multiple challenges such as congestion avoidance, vehicle energy, and end-to-end delay is desired. In this paper, we propose a routing algorithm called TMR used to reduce the time taken by the packets to transmit from source to destination.

## 3. Proposed Protocol

### 3.1. Problem Statement

VANETs use pre-existing proactive and reactive routing protocols like ad hoc on-demand distance vector (AODV), destination-sequenced distance-vector (DSDV), dynamic source routing (DSR), and optimized link state routing (OLSR) [6]. Path selection in these protocols is based on various parameters such as hop count, end-to-end reliability, energy, etc. The major problem in data routing among vehicles is the link failure as a result of node mobility, particularly with high speeds. It is desired to propose a protocol that considers this mobility problem and also to avoid the network congestion to alleviate the traffic load in the network and therefore enhance its performance.

### 3.2. Proposed Solution

In this paper, we present our time delay-based Multipath Routing Protocol named TMR running in a VANET environment. TMR utilizes the RTT measurement to select the shortest path rather than using the minimum number of hops. If one path fails, an alternative path with the next least RTT is used to transfer data messages. TMR sets a threshold value that caps a measured instant RTT to consider a potential route. This threshold is the average RTT that is set to avoid overwhelming the network with data packets when RTT is large or when nodes’ mobility is high. Our protocol is designed to differentiate the emergency and normal message. Emergency messages are sent to the destination without delay, whereas normal messages are pushed in the queue following the FIFO queuing mechanism to reach the destination using TMR protocol.

### 3.3. System Model

For a better understanding of the routing protocol, we first explain the system model as follows:

The node-set of *N* is constituted by the vehicles-set of $V=\{v_{1},v_{2},\cdots ,v_{n}\}$ and the RSU-set of $I=\{rsu_{1},rsu_{2},\cdots ,rsu_{m}\}$. The node-set *N* is defined as N=V∪I where V∩I=∅. Therefore, each node can be placed only in one set. The set of wireless links between RSUs and OBS is denoted by $E=\{l_{1},l_{2},\cdots ,l_{k}\}$. The network graph is represented by G(N,E) for all nodes s∈N and for all links (x,y)∈E.

## 4. Methodology

**Figure 1:**
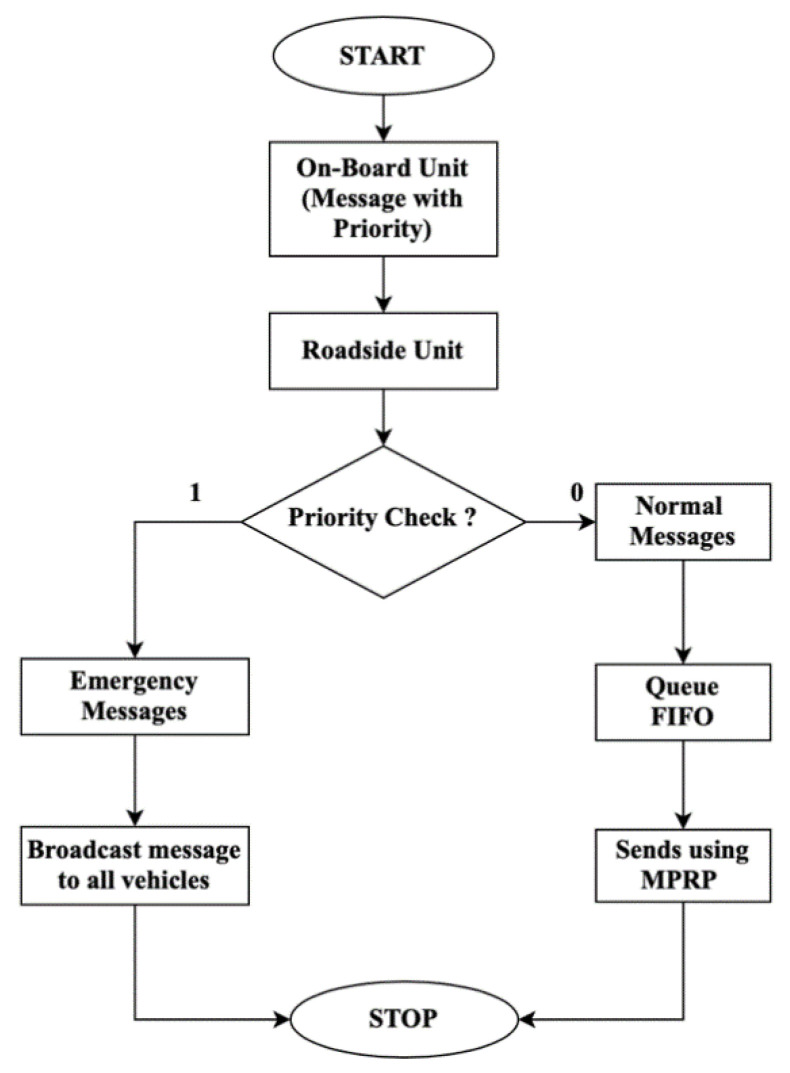
Flow diagram of message handling.

**Figure 2:**
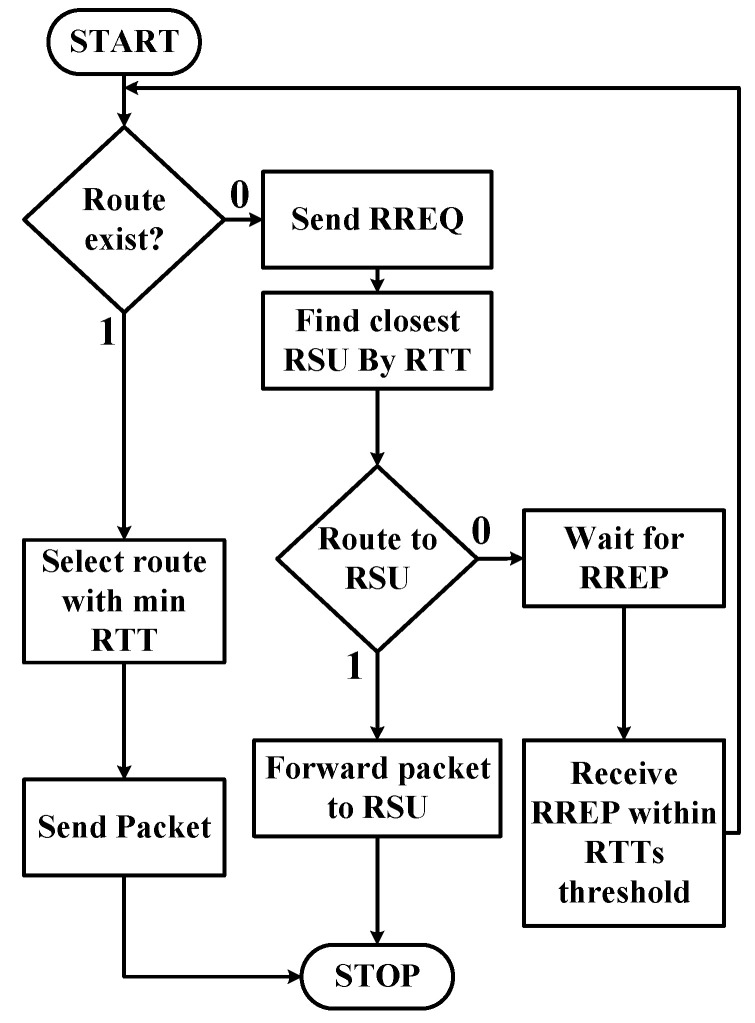
Flow diagram of packet forwarding at OBU.

**Figure 3:**
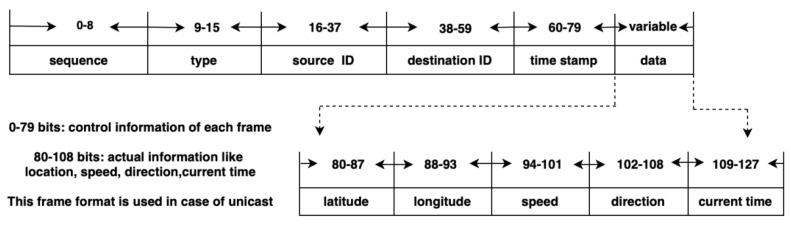
Normal packet format.

TMR is a multipath routing protocol that is based on the RTT that mainly focuses on reducing end-to-end time delay. Figure 1, Figure 2 and Figure 3 show the components used to implement the TMR algorithm using routing path selection, message handling, and RTT concepts. In below, we explain the main components of the message handling in the VANET shown in Figure 1. Figure 2 explains the flow of data transmission at On-Board Unit (OBU).

### 4.1. Methodology Flow

1. OBU: This unit present in the source vehicle is used to send data messages to other vehicles through RSU. Messages produced in the OBU consist of the source vehicle ID (VID) and are assigned a priority value that decides the type of messages.
2. Roadside Unit (RSU): The messages received at RSU are checked for the priority value (0 or 1) to decide which message should be sent first [3]. Therefore, RSU is crucial to control the data traffic in the network; otherwise, every OBU would have access to the whole network and broadcasts an excessive amount of low-priority data packets. Consequently, RSU alleviates traffic problems such as data congestion.
3. Priority Check: As shown in Figure 1, the priority check happens once the messages are received by RSU. If the priority value is 0, then it is considered as a normal message, whereas if the priority value is 1, this is considered as an emergency message and is given the most priority among all the messages received from the OBU.
4. Messages: The messages have information regarding the sender’s vehicle. Based on the content of the message, the priority is determined. These messages are transmitted in the VANETs to disseminate network state or emergency incident information to other vehicles in the network.
  (a) Normal message: Normal message consists of general information about the sender’s vehicle such as the speed of the vehicle, the time at which the message is sent, direction, and location of the vehicle. This information will allow vehicles to get more information about the status of the road in the sense of its crowdedness and hence avoid accidents. The normal messages are sent through unicast communication and are given less priority. These messages are pushed into the queue following the FIFO mechanism to be transmitted to the destination using TMR.
  (b) Emergency message: This message type is time sensitive so it is given a higher priority compared to the normal message because it has the information regarding emergencies (i.e., a collision of vehicles or functional disorder of the vehicle which leads to traffic problems). This message is sent to alert vehicles about such emergencies and, therefore, vehicles should reduce their speed to avoid making such emergency incidents worse particularly on slippery roads. These messages are immediately transmitted among all the vehicles in the network within the RSU range without any delay. So, the approaching vehicles in that range can avoid traffic jams and advance to take a detour.
5. Broadcast: The emergency messages received by RSU are broadcast immediately to all other vehicles in the network [35]. These are alert messages aimed to provide road safety for vehicles of any possible risk.
6. Queue: Queue consists of a list of normal messages to be transmitted sequentially following FIFO.
7. Routing Protocol (TMR): This protocol was implemented to transfer the messages from the source to the destination. The normal message goes through routing protocol which uses the path with the minimum RTT among multiple paths generated. Alternative paths can be used in case there is a link failure.

The unicast transmission also happens in some cases where there is no need to interfere with the entire network in the range of an RSU, even if these messages are somewhat urgent. For example, unicast communication is needed when two cars traveling in different streets are about to meet each other in a blind corner [10]. In such a scenario, vehicles cannot see the other side of the intersection due to hindrance caused by high buildings or trees. At such an instance, the source vehicle communicates with the RSU which in turn sends a message to another vehicle in the opposite direction which is likely to collide. Each road segment is equipped with RSU nodes acting as cluster heads. All communications between OBU nodes take place via RSU. In this case, TMR protocol is utilized to avoid this collision for a message from RSU to the other vehicle(s) in danger in a specific road zone. Another scenario is when a vehicle needs to be aware of a front vehicle’s turns and lane change. When the ahead vehicle turns on an indicator to change direction, a message is sent to the behind vehicle to inform it about this direction change to avoid an accident.

### 4.2. TMR Routing Protocol

RSU sends frequent information messages to all vehicles in its range, informing them about its MAC address. If the vehicle joins newly to the network and does not receive such a message promptly, it can solicit through sending ICMP messages. Normal and emergency messages are sent from vehicles to RSU, which in turn would be conveyed to other vehicles. TMR algorithm is used by RSU to send unicast normal messages to vehicles. Communication between RSU and OBU of vehicles is explained below:

1. All vehicles present in the range of RSU send and receive messages that can be either emergency or normal messages such as vehicle accidents, fire alerts, car speed limits, snow alerts, etc.
2. Initially, OBU sends a message to the RSU using the TMR protocol with assigned priority based on the content in the message. If the message is an emergency, it is assigned 1; whereas, the normal message is assigned 0.
3. If the message is for an emergency case, RSU broadcasts to all vehicles, in its range, without any delay.
4. The normal message is pushed into the queue where it follows the FIFO mechanism. RSU sends these normal messages to vehicles in the network using TMR protocol in that RSU range.
5. Once the RSU receives a message from an OBU, it adds the sending vehicle ID (VID) to the certificate revocation list (CRL) if it does not exist.
6. As shown in Figure 2, RSU checks the routing table for the destination vehicle. If the routing table is empty, RSU broadcasts an RREQ message to all vehicles in that RSU range, and it contains a vehicle ID (VID) of the intended vehicle as being its destination address.
7. The intermediate vehicle OBU adds the VID in the RREQ message to its CRL list if it is not there.
8. If the intermediate vehicle OBU has a path to the destination, it returns RREP, and the RSU, in turn, would calculate the $RTT_{ij}$. If there is no path, OBU will send other RREQ messages to other nodes.
9. $RTT_{ij}$ is measured for every request in all the paths and is stored in an array.
10. The average RTT will be calculated using the following formula.
  $$RTT_{ia}=\sum_{i=0}^{n}\frac{RTT_{ij}}{n}$$
  where,
  i= Number of a specific path
  j= Number of requests per path
  n= Total number of requests
  a= Average of RTT
11. Instant round trip time ($RTT_{ij}$) is compared with a threshold value which is the average round trip time ($RTT_{ia}$). If $RTT_{ij}$ is greater than the average $RTT_{ia}$ then drop the route with that instant $RTT_{ij}$.
12. If the instant $RTT_{ij}$ is less than or equal to the average $RTT_{ia}$, the instant $RTT_{ij}$ route will be added into an array. Then the array is sorted in ascending order. Accordingly, the routing table will be updated using Dijkstra’s algorithm.
13. RSU sends the packet using the first minimum RTT path in the array and waits for the acknowledgment from the destination vehicle.
14. If the time out occurs before receiving an acknowledgment, TMR uses the next minimum RTT path to resend the data packets.
15. This loop performs till the packets are successfully delivered to the destination vehicle.

Steps 1 to 4 are summarized in Algorithm 1. Steps 5 to 8 are summarized in Algorithm 2. Steps 9 to 15 are summarized in Algorithm 3.
**Algorithm 1** Message Handling 1:**procedure** 1: At OnBoard Unit 2:    For OBU message: 3:    **if** (type==emergency) **then** 4:        assign weight = 1 5:    **else**  !type==normal 6:        assign weight = 0 7:    **end if** 8:**end procedure** 9:**procedure** 2: At Roadside Unit10:    Check OBU Message11:    **if** (weight == 1) **then**12:        broadcast Message;13:    **else** !weight == 014:        push Message (FIFO Queue)15:        send data message using TMR in Algorithm 316:    **end if**17:**end procedure**

**Algorithm 2** Routing Path Establishment
 1:**procedure** 1 At RSU: Upon receiving a message from a vehicle to be forwarded to another vehicle 2:    Look Up routing table 3:    **if** (path to destination == null) **then** 4:        Produce RREQ (VID) 5:        Broadcast RREQ (VID) to all nodes (vehicles) in the range 6:    **else** 7:        Follow Algorithm 3 8:    **end if** 9:
**end procedure**
10:**procedure** 2 at OBU and RSU: Receiving RREQ message11:    For each RREQ (VID) received12:    **if** (new VID == old VID) **then**13:        Drop RREQ14:    **else**15:        Add new VID to CRL16:    **end if**17:
**end procedure**
18:**procedure** 3 at OBU: Receiving RREQ message19:    **if** (there is a path to the destination) **then**20:        Send RREP to the RSU21:        RTT is measured at the RSU22:    **else**23:        Broadcast RREQ (VID) to other neighboring nodes24:    **end if**25:
**end procedure**



**Algorithm 3** TMR Routing Protocol
 1:at RSU: 2:While (RREP = true) !RREPs received from Algorithm 2 3:**procedure** 1: Calculating average $RTT_{ij}$ 4:    !*RTT_ij_* received from Algorithm 2 5:    $RTT_{ia}=\frac{\sum_{j=1}^{n}RTT_{ij}}{n}$ 6:
**end procedure**
 7:**procedure** 2 Path Selection 8:    **if** ($RTT_{ij}>RTT_{ia}$) **then** 9:        drop $RTT_{ij}$ route10:    **else**11:        add $RTT_{ij}$ into an array A[]12:        sort array A[] in an ascending order13:        update the routing table14:    **end if**15:    Send a message using a route with the minimum RTT in A[]16:    **if** (timeout) **then**17:        send a message using a route with the next minimum RTT in A[]18:    **end if**19:
**end procedure**



Time delay-based Single path Routing (TSR) protocol uses the same concept of the minimum RTT as TMR protocol, but it provides only a single path. In case of having a link failure, this protocol has to restart the route discovery process to find an alternative path in a similar way to the AODV protocol.

OBU would send a message to RSU, which, in turn, will send the message as a broadcast (emergency) or unicast (normal message to a specific vehicle) [10] to another vehicle OBU. Figure 3 shows the frame format in the case of unicast transmission used with normal messages. The format mainly consists of six categories: Sequence, Type, Source ID, Destination ID, Timestamp, and Data.

1. Sequence number: It is the frame number that helps to avoid redundancy.
2. Type: This indicates the type of a message, i.e., emergency or normal message.
3. Source ID: This contains the sender’s VID to inform the destination (OBU or RSU) from which ID the message is received, an acknowledgment is to be sent.
4. Destination ID: This contains the VID of the destination (OBU or RSU) to which the message should be sent.
5. Timestamp: It can be used in the priority ordering in the FIFO in case of normal messages. In addition, it can be used to reflect status, such as congestion on the road.
6. Data: This field holds the contents of a frame-like latitude, longitude, speed, direction, and the current time.
  (a) Latitude and Longitude: This field holds the exact location of the vehicle with specific latitude and longitude.
  (b) Speed: This field holds the speed of the sender’s vehicle when the message is sent.
  (c) Direction: This field displays the direction in which the sender vehicle is moving.
  (d) Current Time: It displays the time at which the message is sent.

Messages are transmitted using VID as the MAC sublayer addresses. TMR is used in the case of unicast transmission from the RSU to the OBU of a vehicle.

### 4.3. TMR Routing Characteristics

Our algorithms filter those available paths to the final destination in which the instant RTT of a path should be less than or equal to the average RTT for a given route request. If this condition is not met for all available paths, this means that the data packets have no efficient route to the final destination. Therefore, the RSU has to wait for a backoff time and later try again to find a route when the destination vehicle is closer. In this way, excessive data traffic, particularly normal messages, can be reduced to mitigate the pressure on the network and therefore avoid network congestion.

Vehicles’ mobility might harm the precision of the RTT calculation in terms of its aberration, and this is one of the limitations of the algorithms that use RTT. Several techniques utilize RTT in various data communications systems as discussed in [36]. RTT variation relies on the communication model, whether being short-term or long-term transmissions. In VANETs, data communication between RSU and OBU is usually for a short term, where RTT will be updated quite often. This accordingly avoids the RTT inaccuracy happening when vehicles have high speed on the road. In [37], the effect of mobility speed has a limited effect on the RTT calculation as the data packets carrying information can travel back and forth at speeds that are much higher than the node speed. This is valid, particularly in small networks having a shorter path for data packets to traverse. On the other hand, and as in [38], RTT increases with wider networks as having more nodes in the network increases the queuing delay, and this, in turn, would reduce the data packets’ transmission speed. In this case, the data packet traveling speed will be comparable with the vehicle speed, and as a result, RTT accuracy will reduce. To overcome such constraints, it is recommended to have more RSUs in crowded regions such as urban areas to have less distance where packets have to travel, and therefore RTT will reduce. On the contrary, in a high way, particularly between cities, having fewer RSUs can work efficiently as the number of vehicles is relatively small. Therefore, RTT will be short, which in turn increases the data packets’ transmission speed compared to the vehicle speed. RTT can be calculated through a few ICMP echo requests and response packets between the source node (RSU) and the destination node (vehicle) as suggested in [39].

## 5. Experiment Setup and Performance Analysis

To evaluate the performance of TMR, we used NS-3 (version 3.30.1) on Ubuntu 20.04.2 LTS operating system to obtain simulation results. Performance is measured by quantitative metrics of end-to-end delay, packet delivery ratio, packet loss ratio, throughput, and routing overhead. During the simulations, we considered different network configurations with randomly distributed vehicular nodes in a square shape connected roads with 1 km length bidirectional double lane in each direction. Different network loads in terms of having various packet sizes, number of concurrent connections, and different environmental configurations, including mobility speed, number of nodes, and more, are considered to scrutinize the network’s performance. Table 1 shows the simulation parameters. Experiments are all set to parameter assumptions in Table 1 unless otherwise noted. The results indicate the efficiency of our protocol, even as the number of nodes increases.

As TMR uses stationary RSU nodes in its network topology, there was an effort to implement a fair simulation environment for all the protocols. As the result of the same consideration, there was the same number of stationary nodes at the same position in the network for all the simulations.

### 5.1. Performance Metrics

Hereafter, we present the formulas used to measure the performance metrics [11,40]:

1. End-to-end Delay (E2E): End-to-End Delay refers to the time taken by the packet to be transmitted across the network from source to destination. It is generally represented in seconds.
  (1) $$E2E\;\left(\mathrm{in}\;\mathrm{Seconds}\right)=\frac{\sum_{i=1}^{n}\left(R_{i}-S_{i}\right)}{n}$$
  The $R_{i}$ represents the simulator time at the receiving end when the packet delivered. Accordingly, $S_{i}$ represents the time on which the packet is sent out, and, finally, *n* is the number of packets delivered successfully.
2. Packet Loss Ratio (PLR): Packet Loss Ratio is the percentage of packets that failed to deliver to the destination end by a total number of packets sent.
  (2) $$PLR\;\left(\mathrm{in}\;\%\right)=\frac{\sum P_{l}}{\sum P_{g}}\times 100$$
  where $P_{l}$ denotes the number of lost packets and $p_{g}$ represents the number of generated packets.
3. Throughput: Throughput is the number of packets successfully received by all nodes in the unit of time and is represented in Mbps
  (3) $$G\;\left(\mathrm{in}\;\mathrm{Mbps}\right)=\frac{\sum B_{r}\times 8}{T}\times 10^{-6}$$
  In the given equation, *G* represents the throughput, $B_{r}$ is the total number of bytes delivered successfully, and *T* is the time network that has been engaged in data transmission.
4. Routing Overhead (RO): It is the number of routing packets required for network communication that is measured as a percentage (%).
  (4) $$RO\;\left(\mathrm{in}\%\right)=\frac{R_{p}}{R_{p}+D_{P}}\times 100$$
  where $R_{p}$ is the number of routing packets and $D_{p}$ is the number of data packets delivered successfully.
  Routing overhead reveals the amount of control required for the protocol to work. It should be noted that the control packets impose a cost to the network as time, energy, and occupy medium; therefore, more control packets can impact network performance.
5. Energy consumption (EC): The total amount of energy is being used during simulation by all nodes despite their state.
  (5) $$EC\;\left(\mathrm{in}\;\mathrm{Joules}\right)=\sum_{i=1}^{n}\left(I_{i}-E_{i}\right)$$
  where, $I_{i}$ is the initial existing energy of node *i*, $E_{i}$ is remaining energy of node *i* at end of simulation, and *n* represents number of nodes

### 5.2. Results of Scenario 1

**Figure 4:**
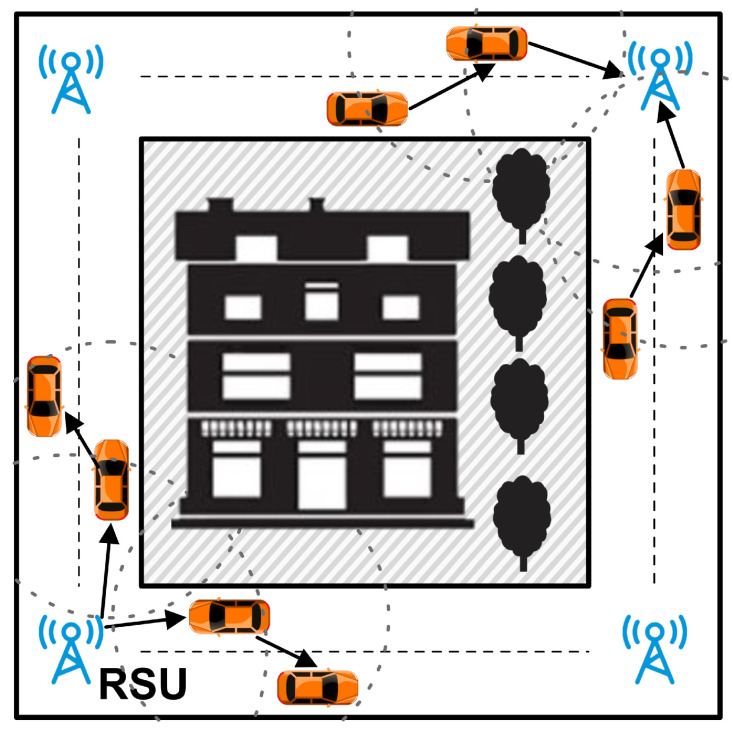
Scenario 1: data communications in one road segment.

In the scenario depicted in Figure 4, we considered a situation in which data communication occurs between OBUs and the RSU close to it in one road segment. The study’s objective in this scenario is to evaluate the effectiveness of each protocol within a road segment. Below are the simulation results of the TMR protocol when compared with AOMDV, TSR, FF-AOMDV, QMR, and EGSR.

#### 5.2.1. Assessment of the Simulation Results Based on Simulation Time

With simulation time progression, more data communications among nodes take place and this, in turn, will increase the data traffic problems such as data congestion and collision. Accordingly, the performance of the network will degrade with the simulation time, as we see in the results below.

**Figure 5:**
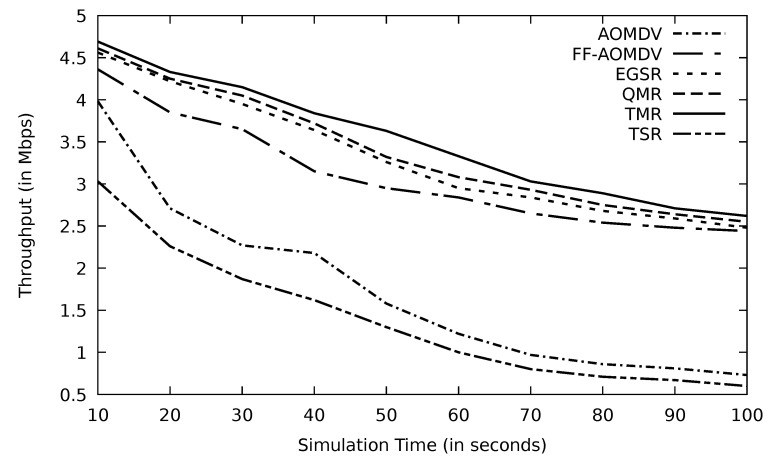
Comparison of AOMDV, FF-AOMDV, EGSR, QMR, and proposed protocol TMR and TSR throughput with simulation time.

In Figure 5, TMR enhances the throughput compared to other protocols. TMR selects the route with the minimum RTT and, therefore, can deliver data to the destination vehicle quicker than other protocols. Furthermore, the minimum RTT implies that the selected route has less traffic and therefore avoids network congestion. In this case, other protocols provide multipath routes, so alternative routes are available. AOMDV selects the shortest route based on less hop count, while FF-AOMDV concentrates more on the path with the highest residual energy rather than the path with less time to reach the destination node as TMR does. TSR performance is poor in comparison with other protocols because it is a single-path protocol, so whenever the link fails, they follow the same route discovery procedure to find a route. Table 2 represents the corresponding value for Figure 5. It also shows how the TMR’s throughput improved in comparison with other protocols.

**Figure 6:**
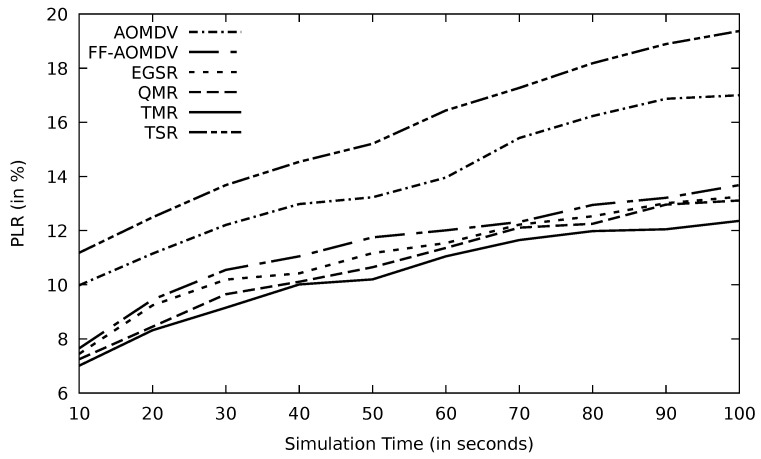
Comparison of AOMDV, FF-AOMDV, EGSR, QMR, and proposed protocol TMR and TSR for PLR with simulation time.

In Figure 6, TMR has a better PLR compared to other protocols. TMR can deliver more data successfully to the destination vehicle, so according to Equation (2), PLR improves. TMR uses the threshold mechanism shown in Algorithm 3, where the optimum routes have less RTT than the average RTT. This, in turn, reduces the PLR. EGSR and QMR protocols also consider the time factor in their route selection mechanisms, and that is why their performance is close to TMR. Other protocols do not consider this time factor, so data packets likely take a long time to be delivered, and PLR enlarges. Additionally, multipath protocols, including TMR, behave better than TSR, particularly when a link failure occurs. TSR has to re-compute the shortest path once there is a link failure, and this, in turn, would increase the processing time that flares up the PLR.

TSR as a single route protocol performs poorly in comparison to the other protocols. Therefore, we eliminate it from other figures to provide more precise results. Similarly, AOMDV has low performance so we remove it from other figures.

#### 5.2.2. Assessment of the Simulation Results Based on Pause Time

Pause time refers to the period in which the vehicle remains stationary. In the following simulation results with pause time, we considered the duration of the red light at the traffic signal as the pause time.

**Figure 7:**
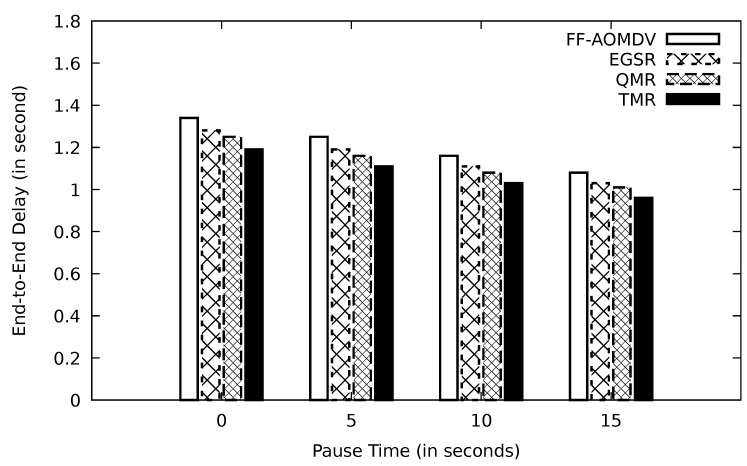
Comparison of FF-AOMDV, EGSR, QMR, and proposed protocol TMR for end-to-end delay with pause time.

In Figure 7, with having a longer pause time, the end-to-end delay decreases. The reasoning behind this incident is that the number of link failures, which is the result of the topological change, has been reduced. With fewer link failure incidents, the time that the sender should be engaged in processing ICMP error messages is shorter. QMR prefers to forward the data packet through the neighbor nodes having mobility speed lower than average speed; therefore, with longer pause time, the performance is improved.

**Figure 8:**
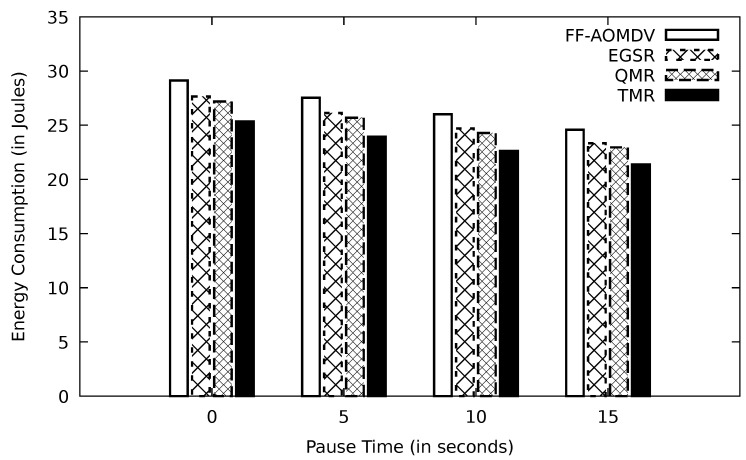
Comparison of FF-AOMDV, EGSR, QMR, and proposed protocol TMR for energy consumption with pause time.

Figure 8 shows that with increasing the pause time, the energy consumption is reduced in all protocols. This is owing to having less chance of a link failure, and the route discovery phase is not often needed. With increasing the pause times, all protocols would send a lower amount of data, and this reduces the needed energy.

#### 5.2.3. Assessment of the Simulation Results Based on Packet Size

In tests of this section, we consider different packet sizes to identify the data load size with the highest throughput. Next, we use that selected packet size in the remaining tests.

**Figure 9:**
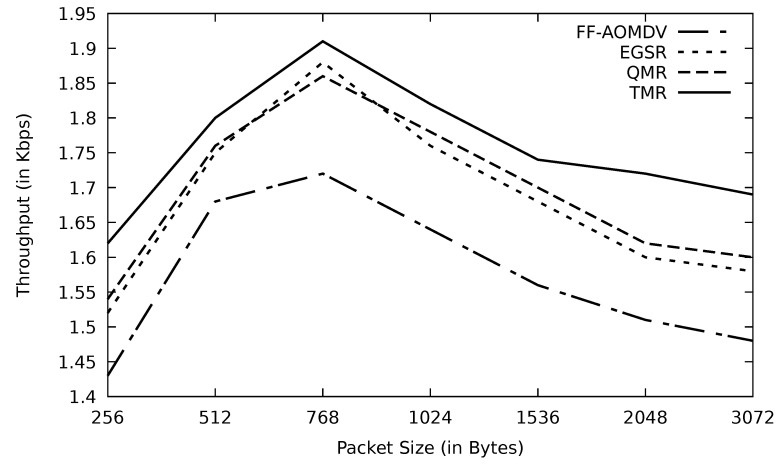
Comparison of FF-AOMDV, EGSR, QMR, and proposed protocol TMR for throughout with different packet sizes.

In Figure 9, the throughput has been calculated based on (3) which shows that the throughput increases with the size of the packets until it reaches 768 bytes. Following that, the throughput decreases as the network starts to suffer from data congestion. However, TMR has the least effect of the congestion on the data transmissions using routes with the minimum RTT, as explained earlier.

**Figure 10:**
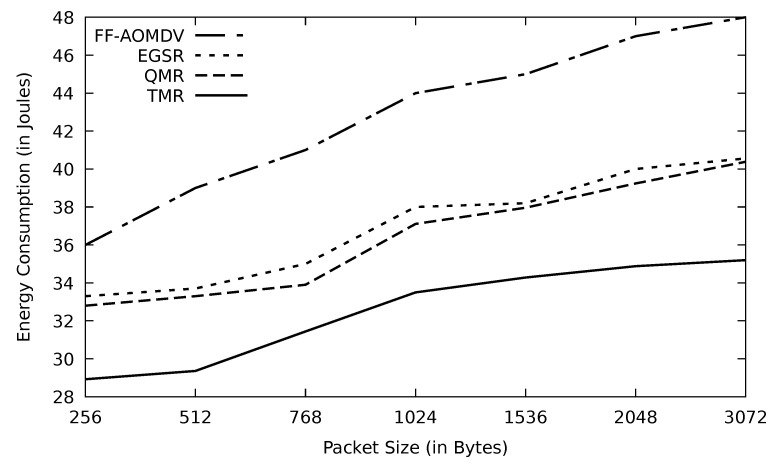
Comparison of FF-AOMDV, EGSR, QMR, and proposed protocol TMR for energy consumption with different packet sizes.

**Figure 11:**
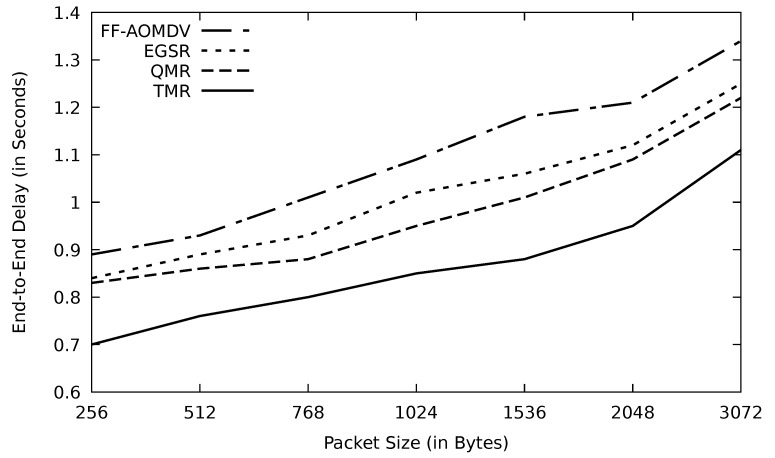
Comparison of FF-AOMDV, EGSR, QMR, and proposed protocol TMR for end-to-end delay with different packet sizes.

As shown in Figure 10, the energy consumption increases when the transmission delay increases as depicted in Figure 11 as a result of the data load size growth. TMR shows the least energy consumption due to its lowest PLR in this simulation. On the other hand, AOMDV shows the worst result, and this is due to having an emerging bottleneck causing a chance of data congestion and collision. The main factor in increasing the end-to-end delay is the queuing time. Avoiding data congestion occurrence is the most promising way to shrink the end-to-end delay. TMR performance in terms of end-to-end delay is high owing to its data traffic problem detection mechanism in terms of RTT threshold.

#### 5.2.4. Assessment of the Simulations’ Result Based on Faulty Node Ratio

**Figure 12:**
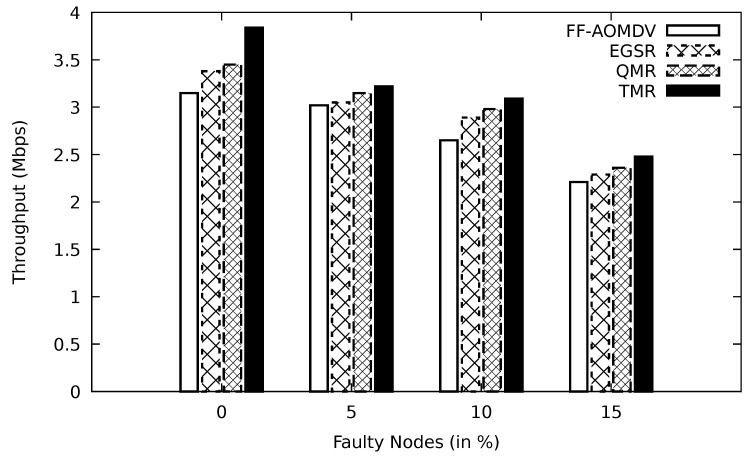
Comparison of FF-AOMDV, EGSR, QMR, and proposed protocol TMR for throughput with faulty node.

We use a threshold value which is the average RTT to determine if the path can be considered in the TMR list of available routing paths. If the instant RTT is higher than the average one, this can be a sign of having traffic congestion in a route, as indicated earlier. In addition, this can be considered as a sign of having faulty nodes that are functioning improperly because of a malfunction of the software or the hardware of the vehicle. The node can fail during the routing discovery phase, and then the route having such a node will be easily dropped. However, the node might fail during the data transmission, so we study this here in our results. In Figure 12, we considered the percentage of faulty nodes to be 0, 5, 10, and 25. As the number of faulty nodes increases, the throughput decreases because of the data loss during the transition period between the routes in the case of multipath algorithms. On the contrary, the transmission will halt during the route discovery phase for each node failure. Minimum RTT involves the minimum number of nodes and the shortest distance, so the effect of a node failure, which results in data loss during transmission, will be less with TMR, and this leads to a higher throughput.

**Figure 13:**
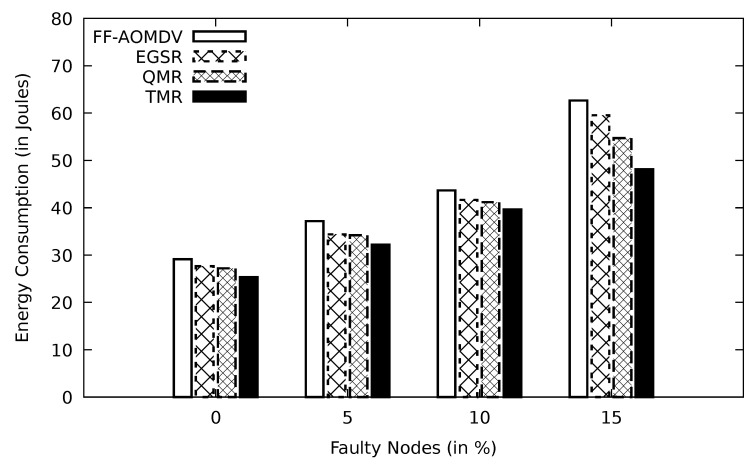
Comparison of FF-AOMDV, EGSR, QMR, and proposed protocol TMR for energy consumption with faulty node.

In Figure 13, increasing the amount of energy consummation is an outcome of the growth in the ratio of faulty nodes. With a higher faulty nodes rate, the PLR increases obviously, and this, in turn, increases the number of data retransmission packets. In this case, the network needs more routing packets to keep the network connectivity. Such extra data loads consume an excessive amount of nodes’ energy.

#### 5.2.5. Assessment of the Simulation Results Based on Number of Nodes

**Figure 14:**
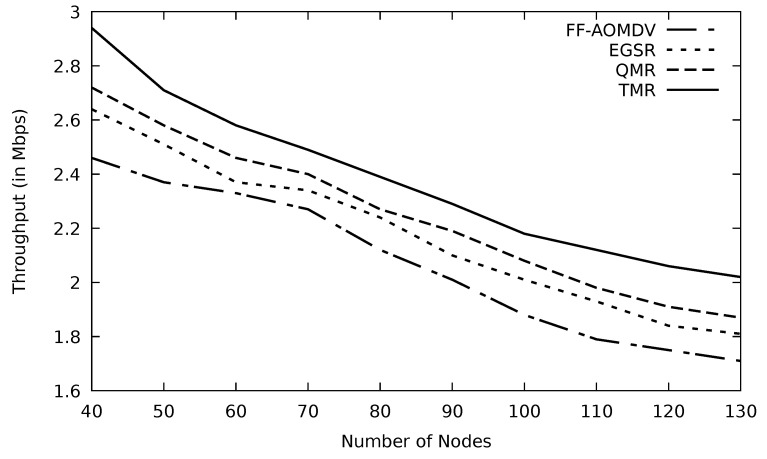
Comparison of FF-AOMDV, EGSR, QMR, and proposed protocol TMR for throughput with number of nodes.

The throughput is high compared to other protocols, as shown in Figure 14. TMR selects the path that has the minimum RTT, and hence it increases the number of packets delivered to the destination node in a given time. Additionally, the route selected based on this mechanism would avoid the congested nodes, as explained above. Table 3 shows improvement of TMR by 14.93, 9.13, and 5.88 percent compared to FF-AOMDV, EGSR, and QMR, respectively.

**Figure 15:**
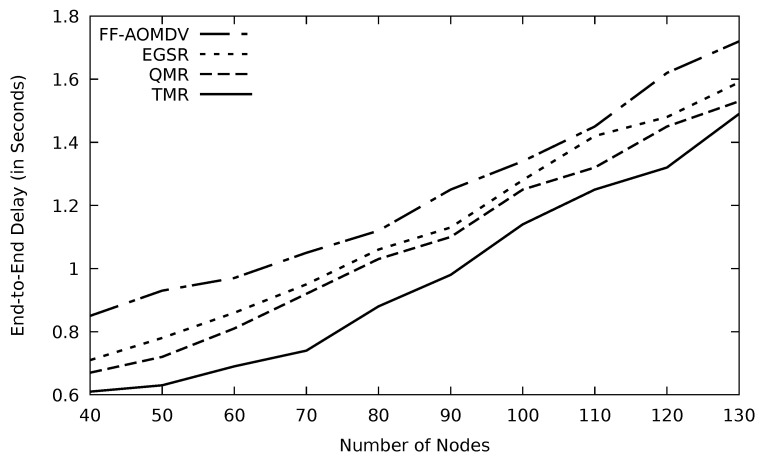
Comparison of FF-AOMDV, EGSR, QMR, and proposed protocol TMR for end-to-end delay with number of nodes.

In Figure 15, increasing the number of nodes as the processing time, to find the efficient route among several possible routes, increases, and therefore the end-to-end delay augments. In the case of multipath protocols, the delay is shorter as alternative paths are always available if a link fails. TMR algorithm can detect and select the least congested link, so the queuing delay will be minimal, and as a result, the end-to-end delay will be relatively short compared to other protocols. The corresponding data is noted in Table 4. TMR reduces the end-to-end delay by 20.9, 13.6, and 9.9 percent compared to FF-AOMDV, EGSR, and QMR, respectively.

**Figure 16:**
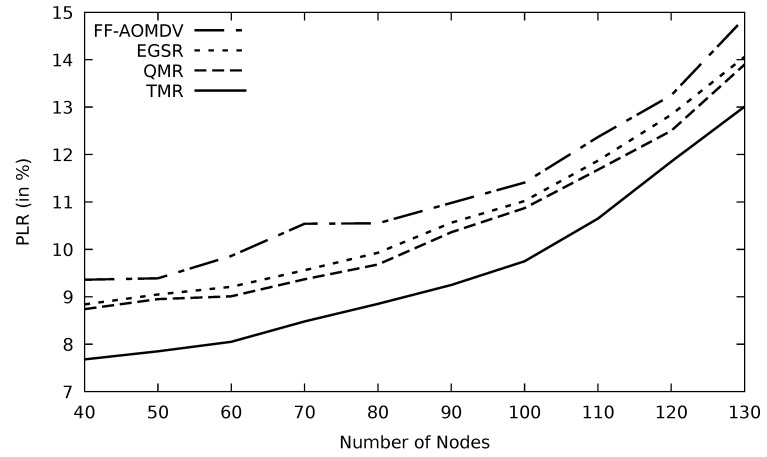
Comparison of FF-AOMDV, EGSR, QMR, and proposed protocol TMR for PLR with number of nodes.

In Figure 16, it is visible that as the number of nodes increases, the number of packets delivered decreases. This is owing to the congested traffic where the obvious amount of data will be dropped and lost. On the other hand, and as indicated earlier, TMR selects the path based on its reliability, so it outperforms other protocols. Additionally, TMR uses the threshold mechanism, and as a result, the packet loss decreases.

**Figure 17:**
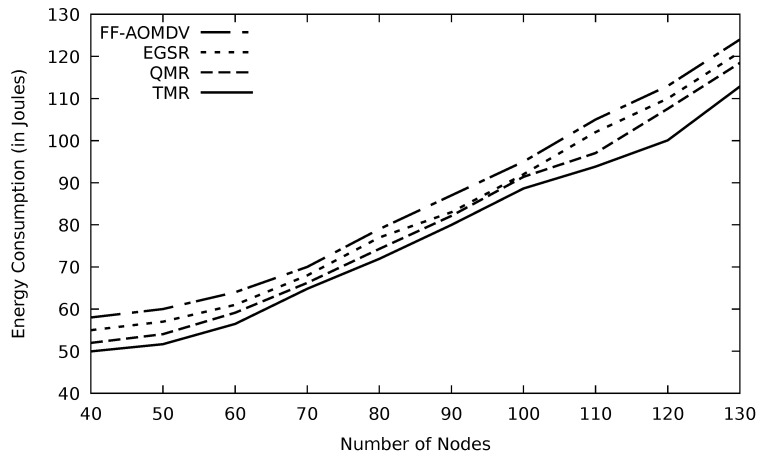
Comparison of FF-AOMDV, EGSR, QMR, and proposed protocol TMR for energy consumption with number of nodes.

A consequence of involving more nodes in data transmission is that a greater amount of energy is going to be used. Every node may receive routing packets and forward them. In this case, the number of neighboring nodes to the sender would increase, and consequently, the energy depletion of the MANET nodes is faster. The result of the energy consumption with the number of nodes is depicted in Figure 17.

#### 5.2.6. Assessment of the Simulation Results Based on Mobility Speed

**Figure 18:**
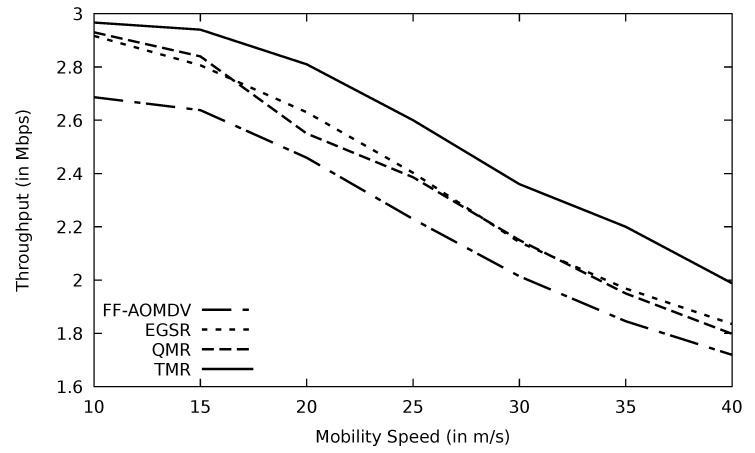
Comparison of AOMDV, FF-AOMDV, EGSR, QMR, and proposed protocol TMR for throughput with mobility speed.

When the mobility speed of the vehicles increases, links incur more instability which in turn would lose more data packets, and this agrees with [41]. Accordingly, fewer packets would be delivered successfully to the destination node, and as a result, the throughput will be diminished. Minimum RTT, utilized in the TMR mechanism, may include less number of nodes and also a shorter distance where packets are traveling. In addition, minimum RTT implies less congested routes where the possibility of having data loss is low, leading to more stable paths. This would minimize the number of nodes that have a high speed in the selected stable route. Therefore, the negative impact of vehicle mobility on the route stability is reduced, and this accordingly enhances the throughput of the TMR protocol compared to other protocols as shown in Figure 18. At speed zero (non-moving vehicles such as parked ones), TMR has a better throughput than other protocols as well as when the mobility speed is higher.

**Figure 19:**
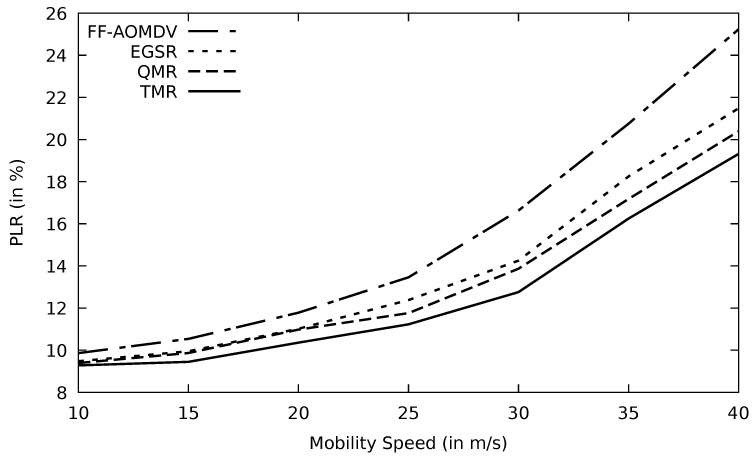
Comparison of FF-AOMDV, EGSR, QMR, and proposed protocol TMR for PLR with mobility speed.

**Figure 20:**
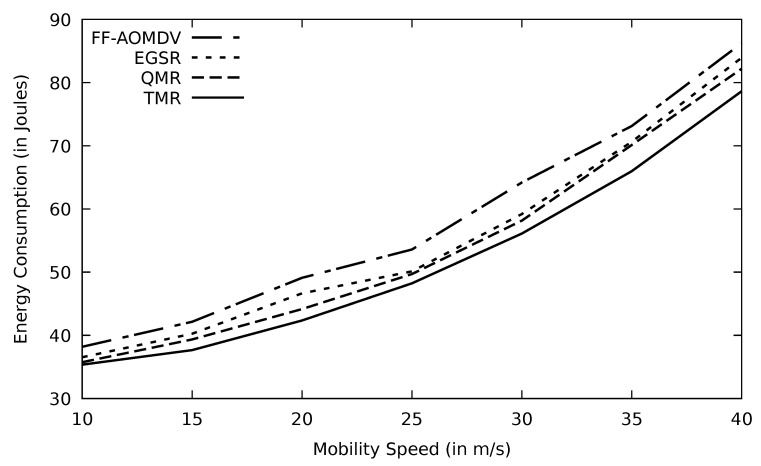
Comparison of FF-AOMDV, EGSR, QMR, and proposed protocol TMR for energy consumption with mobility speed.

In Figure 19, PLR for different protocols performs similarly as throughput versus mobility speed. At 40 m/s (144 km/h) speed, TMR loses just about 13% out of the sent packets, which are still within the accepted range compared to other protocols. The stability of the selected route in terms of packet loss is vital, particularly with link failure caused by node mobility and this is achieved in the TMR algorithm. Additionally, AOMDV delivers about 65% of its data loads to the destination end when mobility speed increases to 40 m/s which is the result of topological inconsistency. At the same mobility speed, AOMDV’s PLR is about 14% higher than TMR. Energy consumption is a metric impacted by PLR. As the nodes’ mobility speed increases, the PLR enlarges, resulting in more data retransmissions. This, in turn, would consume more energy as shown in Figure 20.

**Figure 21:**
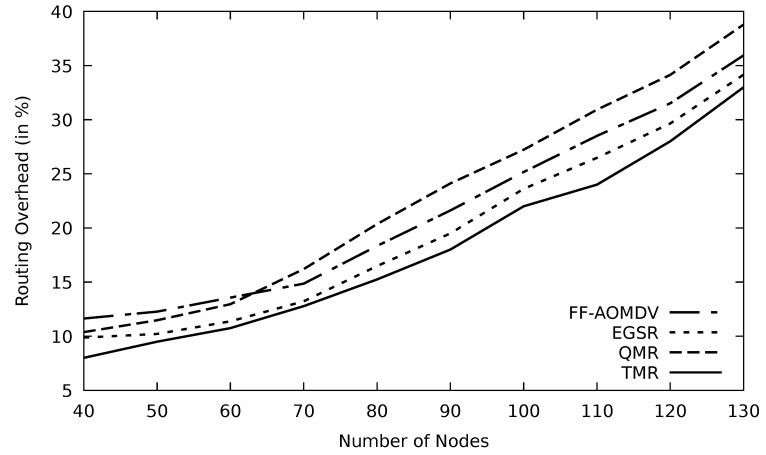
Comparison of FF-AOMDV, EGSR, QMR, and proposed protocol TMR for routing overhead with mobility speed.

**Figure 22:**
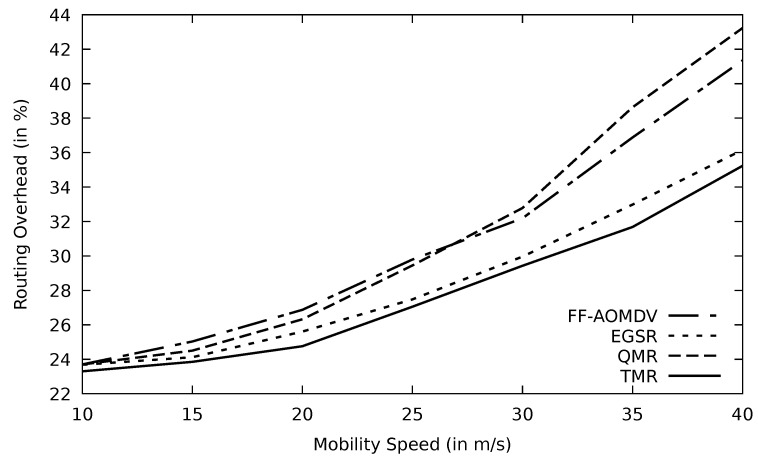
Comparison of FF-AOMDV, EGSR, QMR, and proposed protocol TMR for routing overhead with mobility speed.

Figure 21 and Figure 22 show the routing overhead for different protocols. These protocols seek to find alternate routes in the case of having link failure or faulty nodes to reduce end-to-end delay and increase packet delivery rate. FF-AOMDV routes are calculated based on less energy, so it will be updating its routes quite often, and this, in turn, increases the number of overhead packets. TMR has the least routing overhead among those multipath protocols. RSU sends RREQ to all nodes in its range, and once the RREP is received, routes are determined based on the minimum RTT. Therefore, the control packets are limited, and hence the routing overhead is relatively low.

### 5.3. Results of Scenario 2

**Figure 23:**
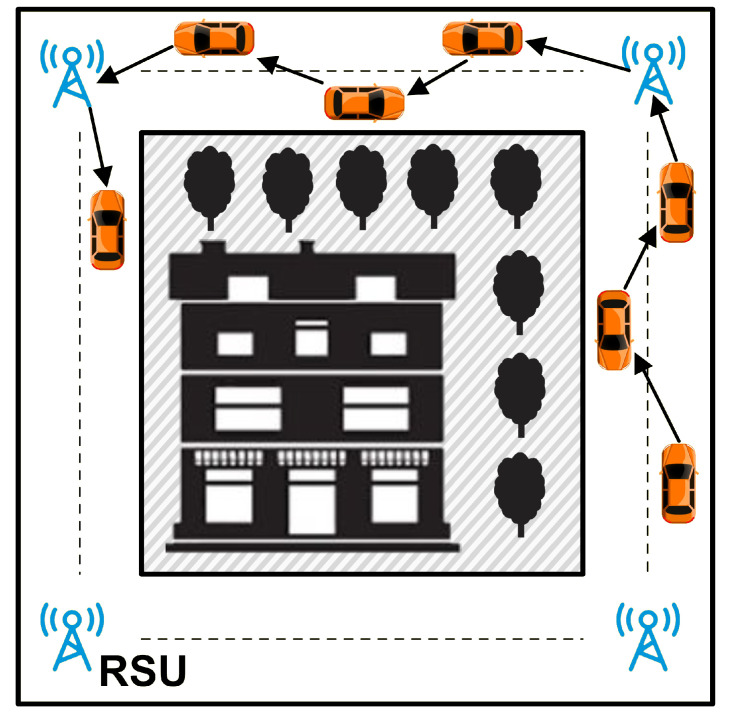
Scenario 2: data communications in multiple road segments.

In the following simulation, the sender and receiver OBUs are located in different road segments; therefore, there is no pair of sender and receivers whose road segments are the same. An exemplification of the given scenario is provided in Figure 23. Simulator selected sender and receiver nodes randomly. ISR algorithm was designed to work appropriately for the given scenario; therefore, we compare QMR and ISR with TMR in this scenario.

#### 5.3.1. Assessment of the Simulation Results Based on Simulation Time

**Figure 24:**
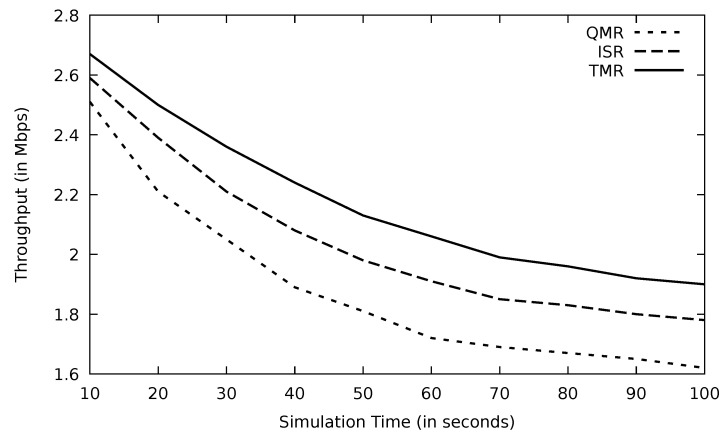
Comparison of EGSR, ISR, and proposed protocol TMR for throughput with simulation time.

**Figure 25:**
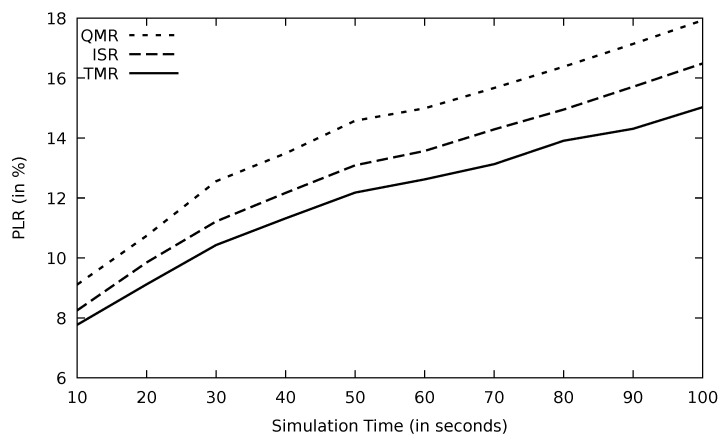
Comparison of EGSR, ISR, and proposed protocol TMR for PLR with simulation time.

The performance of all the protocols drops in terms of throughput as simulation time goes ahead in Figure 24. TMR shows the highest performance as a result of selecting the efficient route based on the minimum RTT in the TMR algorithm and avoiding links with high traffic congestion; this leads to such promising performance. According to Table 5, TMR improves throughput by 15.4 and 6.4 percent compared to QMR and ISR, respectively. Similarly, TMR’s PLR is the lowest in Figure 25, proving its novel performance. The threshold mechanism of TMR in Algorithm 3 selects routes whose RTTs are lower than the average RTT value. Hence, lengthy routes are dropped and therefore, the possibility of having link failure is reduced.

**Figure 26:**
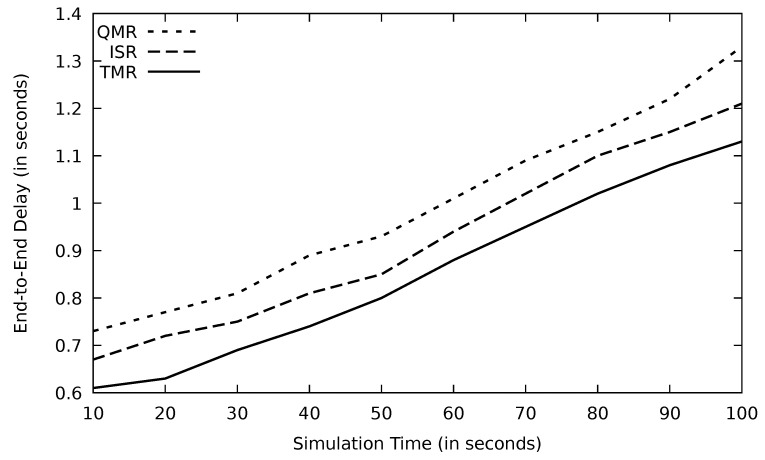
Comparison of EGSR, ISR, and proposed protocol TMR for end-to-end delay with simulation time.

Through time, the network gets more congested, and consequently, the end-to-end delay increases which is the case in Figure 26; however, TMR has the lowest delay due to its route selection mechanism.

**Figure 27:**
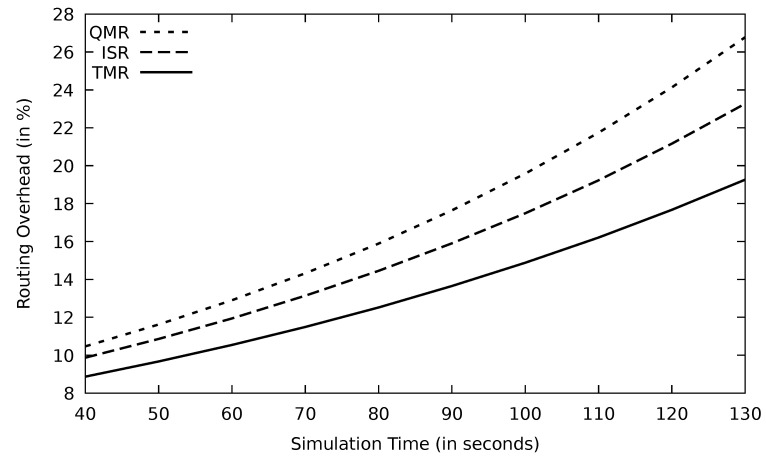
Comparison of EGSR, ISR, and proposed protocol TMR for routing overhead with simulation time.

The routing protocol needs to go through the route discovery phase when the PLR increases. TMR has the lowest PLR and therefore, the route discovery phase is initiated at a lower rate than other protocols. This in turn reduces the routing overhead ratio as shown in Figure 27. Additionally, an excessive amount of routing packets are sent to collect information about the network status such as head nodes selection in case of ISR and the next node in case of QMR. These routing packets are not used in the TMR and, therefore, QMR and ISR have worse routing overhead performance than TMR.

#### 5.3.2. Assessment of the Simulation Results Based on Mobility Speed

**Figure 28:**
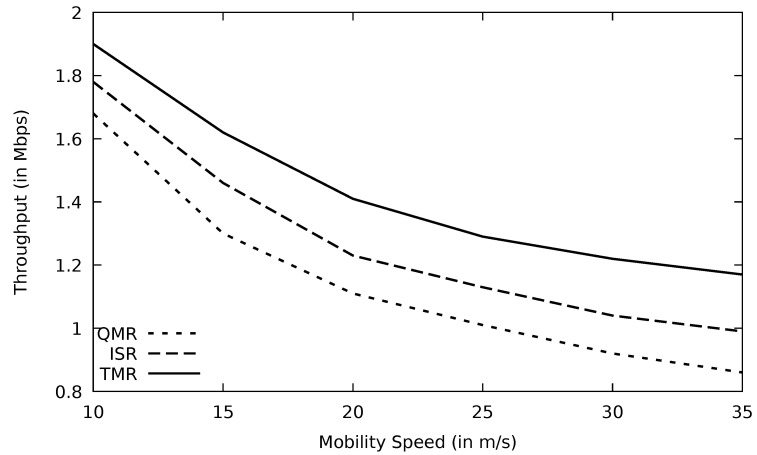
Comparison of QMR, ISR, and proposed protocol TMR for throughput with mobility speed.

**Figure 29:**
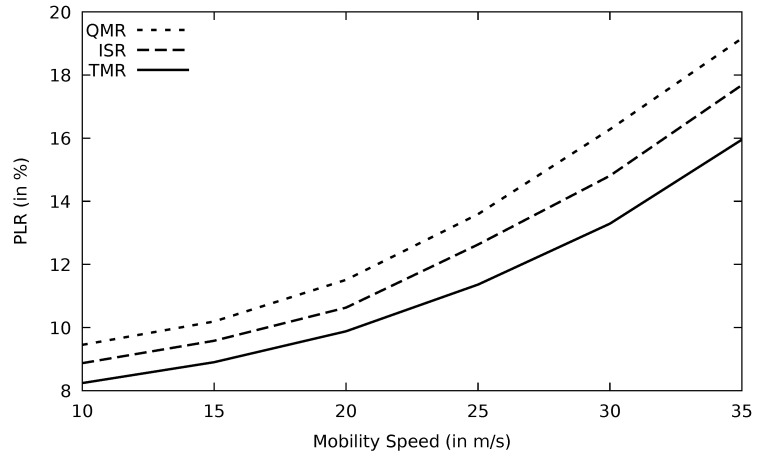
Comparison of QMR, ISR, and proposed protocol TMR for PLR with mobility speed.

The network topology is prone to change when the mobility speed increases. Consequently, the performance of protocols drops, as it is depicted in Figure 28 and Figure 29. When vehicles move at high speed, channels disconnect, and PLR increases. RSU has a wider transmission range than OBU and, therefore, RSU can find more alternative routes using TMR than OBU.

**Figure 30:**
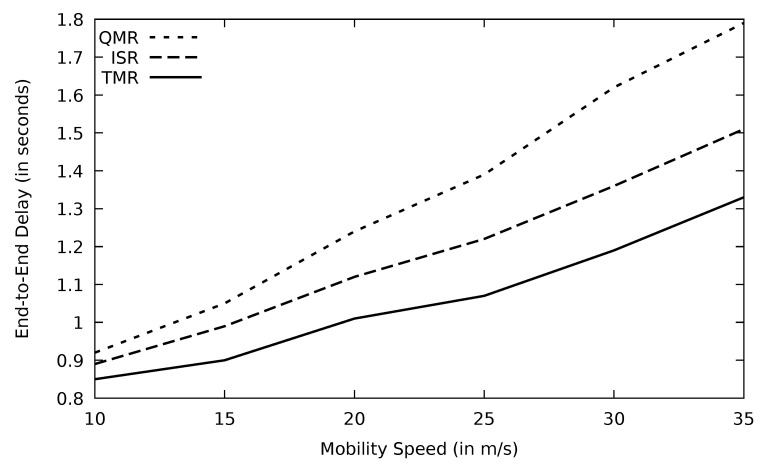
Comparison of QMR, ISR, and proposed protocol TMR for end-to-end delay with mobility speed.

The average end-to-end delay increases when mobility speed increases due to link failure and retransmission of data. The given justification is proven in Figure 30. As the result of the route selection mechanism based on the minimum RTT in TMR, the lowest end-to-end delay emerged for TMR, followed by ISR and QMR. Table 6 indicates that TMR saves end-to-end delay by 26.1 and 11.7 percent compared to QMR and ISR, respectively.

**Figure 31:**
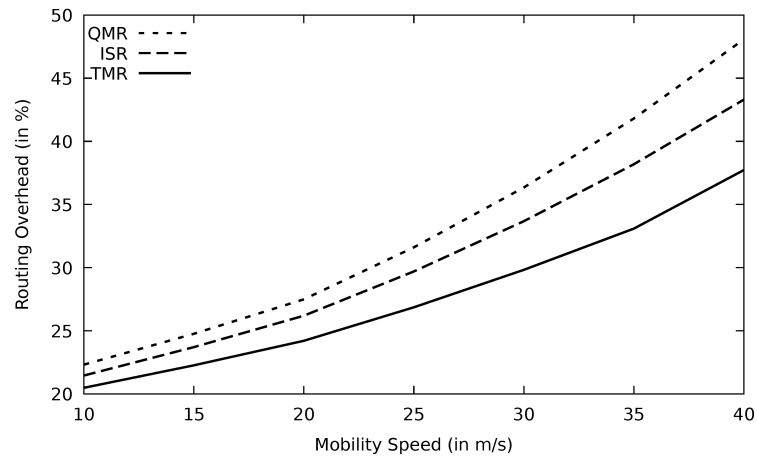
Comparison of QMR, ISR, and proposed protocol TMR for routing overhead with mobility speed.

According to Equation (4), the routing overhead is the result of two parameters: data and routing packets. Therefore, when the PLR increases, the number of successfully delivered data packets drops. Therefore, the routing overhead ratio increases. Additionally, the routing protocol has to go through the routing discovery phase in case of link failure, which produces more routing packets. The TMR has the lowest routing overhead in Figure 31, which proves the throughput and PLR result. With increasing mobility speed, the ISR and QMR routing overhead is distinguishable from TMR.

#### 5.3.3. Assessment of the Simulation Results Based on Number of Nodes

**Figure 32:**
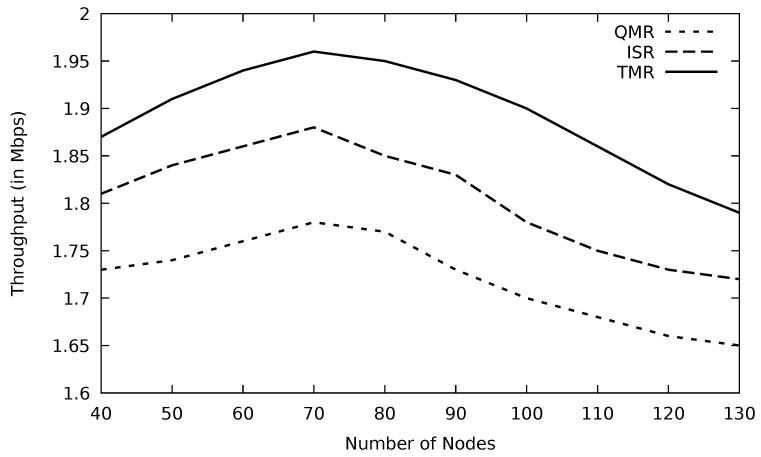
Comparison of QMR, ISR, and proposed protocol TMR for throughput with number of nodes.

**Figure 33:**
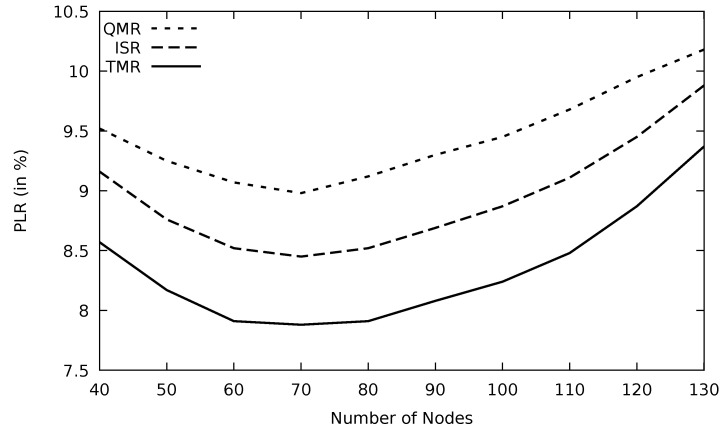
Comparison of QMR, ISR, and proposed protocol TMR for PLR with number of nodes.

The network connectivity depends on the number of nodes in each area. Therefore, when the number of nodes increases, the network is more connected. However, it results in having more data congestion and collision especially when network nodes frequently broadcast emergency messages or cooperative awareness messages (CAM). In Figure 32 and Figure 33, increasing the number of nodes would improve the utilization of the network. This continues until the number of nodes reaches 70, which is the optimum. Beyond that optimum number of nodes, more data packets will result in traffic data jams and hence the performance degrades monotonically. Based on Table 7, TMR degrades PLR by 13.2 and 7.1 percent in comparison with QMR and ISR, respectively.

**Figure 34:**
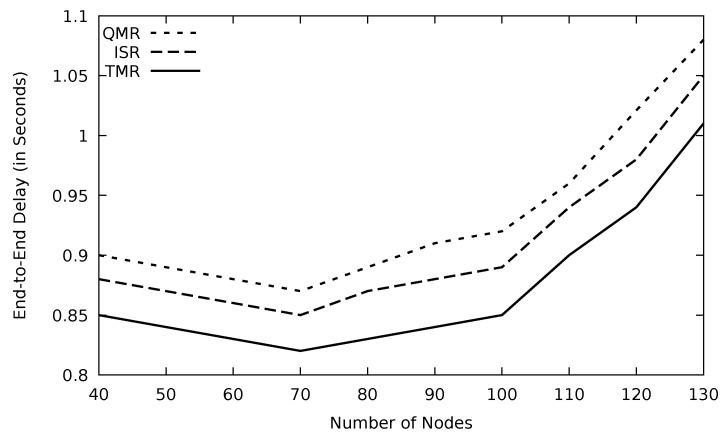
Comparison of QMR, ISR, and proposed protocol TMR for end-to-end delay with number of nodes.

The end-to-end delay increases drastically when the number of nodes goes beyond the optimum value as depicted in Figure 34. The chance of having lengthy routes increases when there are lots of intermediate nodes. TMR mechanism drops those lengthy routes and, therefore, end-to-end delay is minimal.

**Figure 35:**
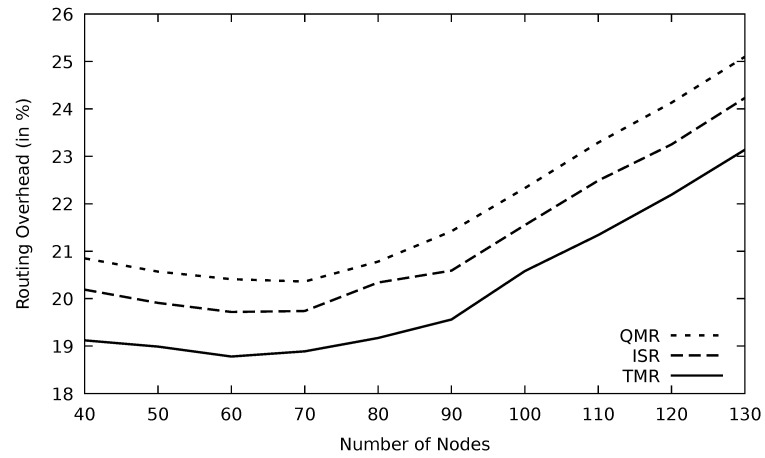
Comparison of QMR, ISR, and proposed protocol TMR for routing overhead with number of nodes.

In Figure 35, the routing overhead increases slightly with the number of nodes because the throughput in this range increases. When the number of nodes is greater than the optimum number, the throughput diminishes as a result of the traffic problems occurrence. In this range, data retransmissions increase and accordingly more routing packets are sent to accommodate those retransmissions.

## 6. Routing Protocol Analysis

In VANETs, energy consumption is not a challenge due to the availability of a significant source of energy. The network nodes could use the vehicle battery. As a result, methods that use energy consumption to measure the traffic condition of the network, such as ETE [42] and FF-AOMDV, are not accurate and do not achieve high performance in VANETs. Moreover, using other methods that rely on successful data delivery probabilities, such as the ETX method, would impose high overhead on the network in terms of sending an excessive amount of link probe packets (LPP) [43]. This, in turn, would add more traffic load to the network, which causes traffic problems such as data packet congestion. On the other hand, RTT measures the status of the network and, therefore, routes can be selected, avoiding those congested paths without the need to add extra overhead. In addition, the route optimization process based on RTT is impressible to frequent topological change in VANETs, which is not the case with using ETX and ETE methods. This feature is more obvious with vehicles having high mobility speeds.

The data path from the sender node to the receiver end may encounter congestion or collision. Algorithms designed based on one-way delay such as [44,45,46,47] rely on the delay only in the reverse path, which may be vulnerable to various traffic challenges compared to the forwarding path. On the other hand, RTT consist of both forwarding and reverse-path delays, and this provides more accuracy. The main difficulty with the routing protocols designed based on the delay to represent the state of the links’ condition is their dependency on the clock synchronization [48]. The clock of all the nodes should be synced to calculate the accurate RTT; otherwise, the performance could be impacted by the accuracy of the calculated RTT.

Another difficulty is the placement of the RSU devices in the network. To optimize the network performance, the number and position of RSU nodes need to be determined precisely. RSU devices play a key role in the network topology, and it is more likely to be placed where there is a physical obstacle to provide steady coverage as well as where vehicles are operating at a high mobility speed [49].

Table 8 provides the required characteristics for each protocol. In this comparison, protocols such as EGSR, QMR, and ISR are dependant on the GPS, which may cause a bigger routing packet size. Additionally, it may impose computation load on nodes. However, TMR does not need to use the GPS and, therefore, the overload in terms of packet size is minimized and this accordingly reduces the traffic load in the network. Several pieces of research do not consider computing energy requirements in terms of energy consumption. With limited resource devices, it could be time and energy consuming. As an exemplification, EGSR considered that each node has sufficient computation resources, which is not always the case. On the other hand, TMR is a reactive routing algorithm and this minimizes the energy consumption as the routes are optimized on-demand. Regarding scalability, road segments in TMR are limited by the average RTT threshold between OBU nodes and RSU nodes. This in turn will reduce the latency and enhance the data throughput. In EGSR, the road connecting two adjacent intersections is considered as the road segment.

### Routing Protocol Time Complexity

**Figure 36:**
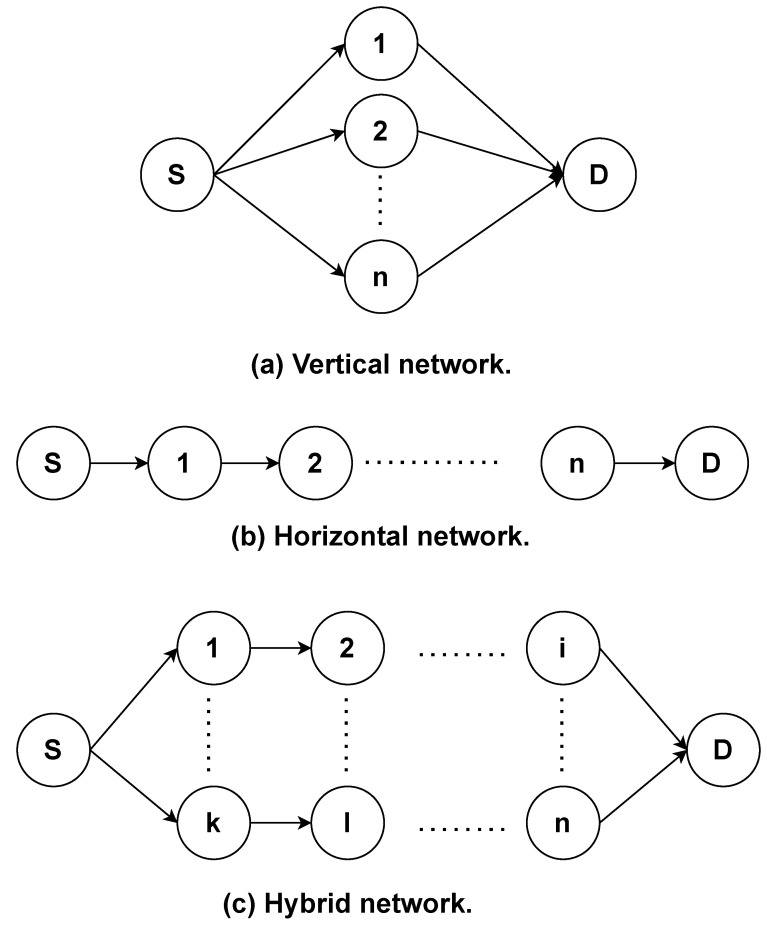
Scenarios of time-complexity networks.

To understand the time complexity of the route discovery phase, we consider three different scenarios as depicted in Figure 36. In this regard, we consider that there is *n* number of intermediate nodes in the network.

According to Scenario (a), all the intermediate nodes are receiving the RREQ packet simultaneously and all of them broadcast it towards the destination D in the worst-case scenario where the route to D is not available in any node. Therefore, the order of RREQ broadcasting sent by source node S is O(n). Furthermore, each intermediate node may rebroadcast RREQ and all other nodes receive that. Therefore, each node will receive RREQ one time from node S and *n* times from other nodes. Therefore, the entire network deals with the time complexity of $O(n^{2})$ on receiving RREQ.

In contrast with Scenario (a), in Scenario (b), the intermediate nodes are arranged serially. Each node receives the RREQ once from the previous node and once from the next node in the worst-case scenario. Therefore, the order of RREQ flooding is O(2n).

As to Scenario (c), if all the intermediate nodes are included in a route, then the time complexity of RREQ remains O(n). Each node will receive an RREQ *n* times from other nodes. Therefore, the entire network deals with the time complexity of $O(n^{2})$ on receiving RREQ.

In conclusion, the number of times that a network must go through the route discovery phase depends on the network configuration and topology. This is the reason we need to study the routing protocols via simulation.

## 7. Conclusions

In this paper, we propose a Multipath Routing Protocol called TMR that makes use of the round trip time (RTT) to perform packet transmission tasks to the destination node in VANETs. The main idea in the TMR routing protocol is the implementation of centralized network intelligence in one component of the network to reduce the time taken while maintaining the consensus between source and destination for efficient packet data transmission. Most of the data communications between vehicles should go through the Road-Side Unit (RSU) to control packets transmission and reduce the data traffic in the network. TMR primarily selects the optimum route that has the minimum RTT, and this implies a route that has the least traffic problems such as data congestion and collision. Optimum routes should have RTT that is shorter than a threshold value which is set as the average RTT. This mechanism secures a short route to the RSU. It hence reduces the possibility of having packet loss that may occur with high-speed mobility vehicles getting further away from the RSU. This, in turn, would speed up the transmission of alert messages between vehicles and therefore lessen vehicle accidents. Moreover, TMR reduces the data retransmissions, and this minimizes the data traffic load to deliver the normal messages promptly. The performance of the proposed TMR is assessed by simulations and compared to other protocols. TMR exhibits an increase in the rate of successfully delivered packets over AOMDV, FF-AOMDV, EGSR, QMR, and ISR, respectively. The overall simulation results show that TMR can greatly improve the performance of data communications in VANETs even as the number of vehicles increases. Specifically, TMR can achieve performance enhancement with 5% to 26% overall comparative protocols. In the future, vehicles’ density and RSUs distribution on the road should be considered in the data routing methods in VANETs.

## Figures and Tables

**Table 1:** Simulation parameters.

Parameter Type	Parameter Value
Network Simulator	ns-3.30.1
Traffic Simulator	SUMO 1.7.0
Wireless Protocol	IEEE 802.11p
MAC and Physical Layer standard	OFDM rate (6 Mbps, 9 Mbps, 12 Mbps, 18 Mbps, 24 Mbps, 36 Mbps, 48 Mbps, 54 Mbps) 20 MHz
Protocols	AOMDV, TSR, FF-AOMDV, EGSR, QMR, ISR, and TMR
Number of runs	5
Simulation Time	10, 20, 30, 40, 50, 60, 70, 80, 90, 100 s
Number of nodes	40, 50, 60, 70, 80, 90, 100, 110, 120, 130
Mobility speed	10, 15, 20, 25, 30, 35, 40 m/s
Number of concurrent Connections	5, 8, 10, 11, 14, 17, 20, 23, 26, 29
Data payload	256, 512, 768, 1024, 2048 3072 bytes/packet
Transmission speed	1024 Kbps
Transmission Power	7.5 db
Initial Energy Source	100 Joules
Transmission Energy	0.2 watt
Receiving Energy	0.1 watt

**Table 2:** Simulation time vs. throughput of Figure 5.

Time	TSR	AOMDV	FF-AOMDV	EGSR	QMR	TMR
10	3.03	3.98	4.36	4.56	4.61	4.69
20	2.26	2.71	3.85	4.22	4.25	4.33
30	1.87	2.27	3.65	3.95	4.05	4.15
40	1.62	2.18	3.15	3.64	3.72	3.84
50	1.3	1.58	2.95	3.26	3.32	3.63
60	1.02	1.22	2.84	2.95	3.08	3.33
70	0.8	0.97	2.65	2.84	2.93	3.03
80	0.71	0.86	2.54	2.68	2.75	2.89
90	0.67	0.81	2.48	2.59	2.64	2.71
100	0.6	0.73	2.44	2.48	2.55	2.62
Sum	13.88	17.31	30.91	33.17	33.9	35.22
Gain (%)	153.74	103.46	13.94	6.18	3.89	

**Table 3:** Number of nodes vs. throughput of Figure 14.

#Node	FF-AOMDV	EGSR	QMR	TMR
40	2.46	2.64	2.72	2.94
50	2.37	2.51	2.58	2.71
60	2.33	2.37	2.46	2.58
70	2.27	2.34	2.4	2.49
80	2.12	2.24	2.27	2.39
90	2.01	2.1	2.19	2.29
100	1.88	2.01	2.08	2.18
110	1.79	1.93	1.98	2.12
120	1.75	1.84	1.91	2.06
130	1.71	1.81	1.87	2.02
sum	20.69	21.79	22.46	23.78
gain (%)	14.93	9.13	5.88	

**Table 4:** Number of nodes vs. end-to-end delay of Figure 15.

#Node	FF-AOMDV	EGSR	QMR	TMR
40	0.85	0.71	0.67	0.61
50	0.93	0.78	0.72	0.63
60	0.97	0.86	0.81	0.69
70	1.05	0.95	0.92	0.74
80	1.12	1.06	1.03	0.88
90	1.25	1.13	1.1	0.98
100	1.34	1.28	1.25	1.14
110	1.45	1.42	1.32	1.25
120	1.62	1.48	1.45	1.32
130	1.72	1.59	1.53	1.49
sum	12.3	11.26	10.8	9.73
saving (%)	20.9	13.6	9.9	

**Table 5:** Simulation time vs. throughput of Figure 24.

Time	QMR	ISR	TMR
10	2.51	2.59	2.67
20	2.21	2.39	2.5
30	2.05	2.21	2.36
40	1.89	2.08	2.24
50	1.81	1.98	2.13
60	1.72	1.91	2.06
70	1.69	1.85	1.99
80	1.67	1.83	1.96
90	1.65	1.8	1.92
100	1.62	1.78	1.9
sum	18.82	20.42	21.73
gain (%)	15.5	6.4	

**Table 6:** Mobility speed vs. end-to-end delay of Figure 30.

Speed	QMR	ISR	TMR
10	0.92	0.89	0.85
15	1.05	0.99	0.9
20	1.24	1.12	1.01
25	1.39	1.22	1.07
30	1.62	1.36	1.19
35	1.79	1.51	1.33
sum	8.01	7.09	6.35
saving (%)	26.1	11.7	

**Table 7:** Number of nodes vs. PLR of Figure 33.

#Node	QMR	ISR	TMR
40	9.52	9.16	8.57
50	9.25	8.76	8.17
60	9.07	8.52	7.91
70	8.98	8.45	7.88
80	9.12	8.52	7.91
90	9.3	8.69	8.08
100	9.45	8.87	8.24
110	9.68	9.11	8.48
120	9.95	9.45	8.87
130	10.18	9.88	9.37
sum	94.5	89.41	83.48
saving (%)	13.2	7.1	

**Table 8:** Routing protocol characteristics comparison.

Characteristics	TMR	EGSR [22]	QMR [16]	FF-AOMDV [11]	ISR [24]	AOMDV
Clock Synchronization	Yes	Yes	No	No	No	No
GPS	No	Yes, required as it is geographic aware protocol	Yes, acquire position	NO	Yes, required for positioning the head node	No
Energy optimization	No	No	Yes	Yes	No	No
Scalability approach	Road segmentation	Road segmentation	No	No	Road segmentation	No
Routing Discovery	Reactive protocol	Proactive protocol, based on $t_{ant}$	Hybrid protocol, Q-learning with variable α and γ	Reactive protocol	Proactive protocol	Reactive protocol
RTT	Yes	NO	NO	NO	NO	NO

## Data Availability

Not applicable.
